# The *Drosophila* orthologue of the INT6 onco-protein regulates mitotic microtubule growth and kinetochore structure

**DOI:** 10.1371/journal.pgen.1006784

**Published:** 2017-05-15

**Authors:** Fioranna Renda, Claudia Pellacani, Anton Strunov, Elisabetta Bucciarelli, Valeria Naim, Giuseppe Bosso, Elena Kiseleva, Silvia Bonaccorsi, David J. Sharp, Alexey Khodjakov, Maurizio Gatti, Maria Patrizia Somma

**Affiliations:** 1 Dipartimento di Biologia e Biotecnologie “C. Darwin”, Sapienza, Università di Roma, Roma, Italy; 2 Wadsworth Center, New York State Department of Health, Albany, New York, United States of America; 3 Istituto di Biologia e Patologia Molecolari (IBPM) del CNR, Roma, Italy; 4 Institute of Molecular and Cellular Biology, Siberian Branch of RAS, Novosibirsk, Russia; 5 Institute of Cytology and Genetics, Siberian Branch of RAS, Novosibirsk, Russia; 6 Department of Physiology and Biophysics, Albert Einstein College of Medicine, Bronx, New York, United States of America; Geisel School of Medicine at Dartmouth, UNITED STATES

## Abstract

INT6/eIF3e is a highly conserved component of the translation initiation complex that interacts with both the 26S proteasome and the COP9 signalosome, two complexes implicated in ubiquitin-mediated protein degradation. The *INT6* gene was originally identified as the insertion site of the mouse mammary tumor virus (MMTV), and later shown to be involved in human tumorigenesis. Here we show that depletion of the *Drosophila* orthologue of *INT6* (*Int6*) results in short mitotic spindles and deformed centromeres and kinetochores with low intra-kinetochore distance. Poleward flux of microtubule subunits during metaphase is reduced, although fluorescence recovery after photobleaching (FRAP) demonstrates that microtubules remain dynamic both near the kinetochores and at spindle poles. Mitotic progression is delayed during metaphase due to the activity of the spindle assembly checkpoint (SAC). Interestingly, a deubiquitinated form of the kinesin Klp67A (a putative orthologue of human Kif18A) accumulates near the kinetochores in Int6-depleted cells. Consistent with this finding, Klp67A overexpression mimics the *Int6* RNAi phenotype. Furthermore, simultaneous depletion of *Int6* and *Klp67A* results in a phenotype identical to RNAi of just Klp67A, which indicates that Klp67A deficiency is epistatic over Int6 deficiency. We propose that Int6-mediated ubiquitination is required to control the activity of Klp67A. In the absence of this control, excess of Klp67A at the kinetochore suppresses microtubule plus-end polymerization, which in turn results in reduced microtubule flux, spindle shortening, and centromere/kinetochore deformation.

## Author summary

*INT6* is an evolutionarily conserved gene originally identified as the insertion site of the mouse mammary tumor virus that causes tumors in mice. *INT6* is downregulated in many human cancers, suggesting that it acts as tumor suppressor gene. The INT6 protein is involved in several biological processes, including translation and ubiquitin-mediated protein degradation. We performed RNA interference (RNAi) against the *Drosophila* homologue of *INT6* (*Int6*) and analyzed the effects of Int6 depletion on mitotic cell division. We found that loss of Int6 results in short spindles, delayed progression though metaphase and abnormally shaped centromeres/kinetochores. We also found that Int6-depleted cells fail to degrade the kinesin Klp67A. This protein, known to attenuate polymerization of microtubule (MTs) plus ends, accumulated at the kinetochores in Int6-depleted cells. We propose that this condition affects MT growth at the kinetochore, which in turn results in centromere/kinetochore deformation and delays satisfaction of the mitotic checkpoint.

## Introduction

The *INT6* gene was originally identified as the insertion site of the mouse mammary tumor virus (MMTV) [1]. MMTV integration into the *INT6* gene causes the production of a C-terminally truncated Int6 protein (INT6ΔC). Ectopic expression of INT6ΔC in mouse mammary glands leads to tumor formation [2]. In addition, INT6ΔC can induce malignant transformation of human tissue culture cells, which produce tumors when injected into immunodeficient mice [2–4]. However, the examination of several breast cancer cell lines did not detect INT6ΔC expression [2,5]. Moreover, many human breast cancers are characterized by INT6 deregulation; some tumors show low levels of INT6 [6–9], while others exhibit an upregulation of the protein [10]. Thus, even if in most cases *INT6* acts as a tumor suppressor, it can also have an oncogenic role.

INT6 is a highly conserved protein that has been also identified as a subunit (eIF3e) of the eukaryotic translation initiation factor eIF3 [11]. INT6/eIF3e interacts with subunits of the COP9 signalosome (CSN) and 26S proteasome, which are involved in protein ubiquitination and degradation of polyubiquitinated proteins, respectively [12–14]. Consistent with these biochemical data, studies carried out in diverse systems have implicated INT6 in the regulation of the three complexes. In contrast to other eIF3 subunits, INT6/eIF3e is not essential for global translation and appears to mediate the translation of a limited subset of mRNAs [5,15–17]. In both fission yeast and humans, INT6 promotes proteasome assembly via its interaction with the Rpn5 proteasomal subunit, and INT6-depleted cells accumulate polyubiquitinated proteins [18]. There is also evidence that INT6 is functionally related with the CSN complex. For example, the *Drosophila* orthologue of INT6 (Int6) regulates CSN-mediated cullin neddylation [19].

INT6 has been implicated in mitotic division in budding yeast, *Drosophila* and human cells. Studies in S. *pombe*, have shown that *Yin6*, the yeast orthologue of *INT6*, cooperates with *Ras1* to ensure proper chromosome segregation. Defective chromosome segregation was rescued by human *INT6*, highlighting the functional conservation of the gene [18,20]. RNAi mediated depletion of INT6 in human cells resulted in abnormal spindles, defective chromosome alignment at metaphase, and failure in cytokinesis, a phenotype attributed to reduced activity of the Cdk1 kinase [21].

Int6-depleted cells have been shown to delay during metaphase with short spindles [22]. Here we demonstrate that in *Int6* RNAi cells spindle shortening is accompanied by a deformation of both centromeres and kinetochores, a reduction of the intra-kinetochore distance, and a persistent inability to satisfy the spindle checkpoint (SAC). Our results suggest that these phenotypic traits are the consequence of an accumulation at kinetochores of a non-ubiquitinated form of Klp67A, a conserved plus-end-directed kinesin-like protein that suppresses microtubule (MT) polymerization at plus ends [23–27].

## Results

### Int6 depletion results in short spindles and affects anaphase chromosome movement

**Fig 1:**
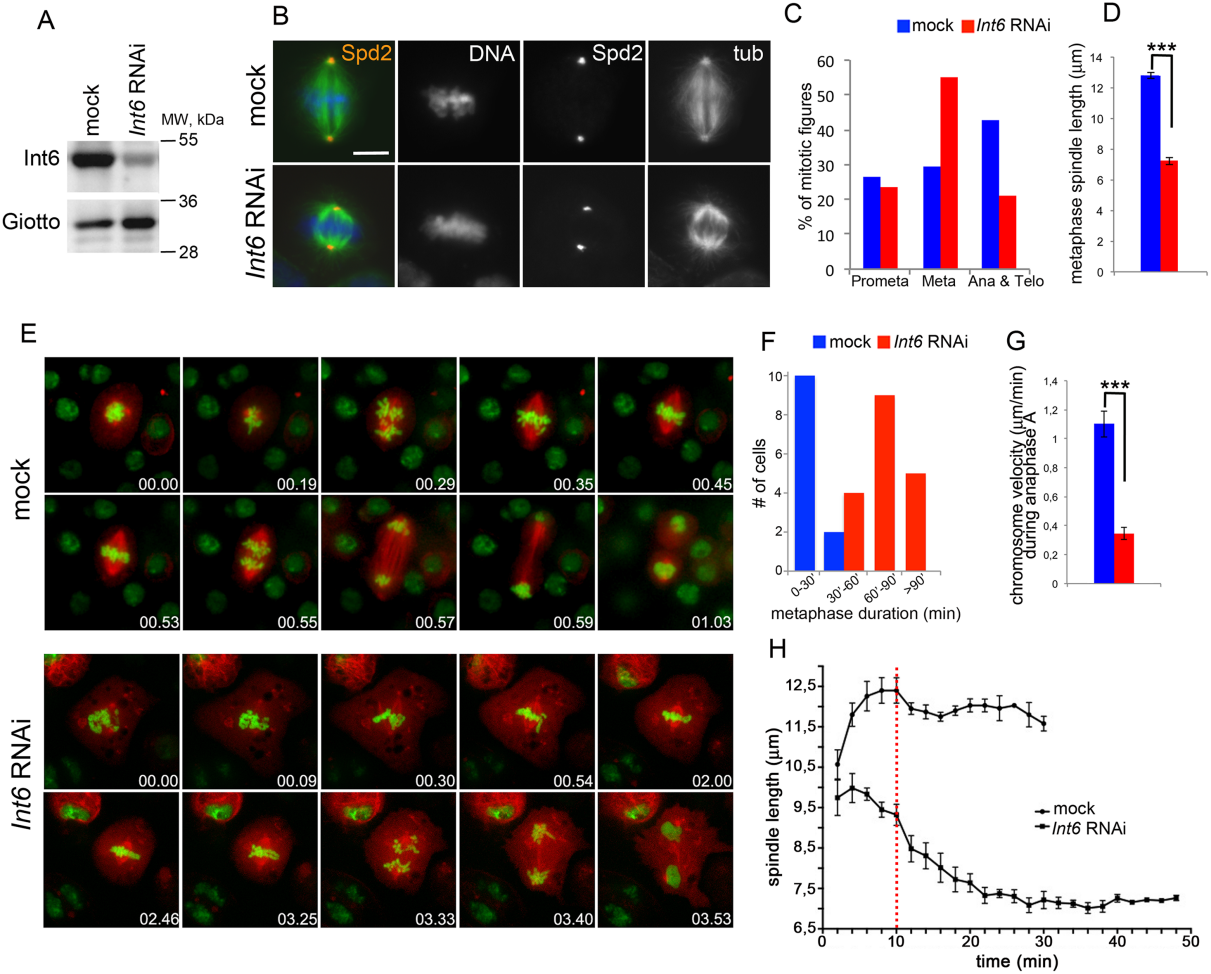
RNAi-mediated depletion of Int6 results in delayed progression through metaphase and spindle shortening. (A) Western blot of S2 cell extracts from control and *Int6* RNAi cells. Giotto, a phosphatidylinositol transfer protein (Giansanti et al, 2006 [62], was used as a loading control. (B) Metaphases from mock-treated and *Int6* RNAi cells stained for DNA (blue), tubulin (green) and Spd2 (red). Scale bar, 5 μm. (C) Frequencies of mitotic figures in fixed *Int6* RNAi cells (n = 443) and mock-treated cells (n = 330). (D) Average metaphase spindle length (± SEM) in *Int6* RNAi cells (n = 80) and mock-treated cells (n = 80). ***, significantly different in the Student’s t test with p < 0.0001. (E) Stills from time-lapse videos of mitosis in mock-treated and *Int6* RNAi cells expressing histone-GFP and mCherry-tubulin. The numbers at the bottom of each frame indicate hours and minutes (hh.mm) elapsed from the beginning of imaging. See also S1 and S2 Movies. (F, G) Metaphase duration (F) and chromosome segregation velocity during anaphase A (± SEM) in mock-treated and *Int6* RNAi cells (G). ***, significantly different in the Student’s t test with p < 0.0001. (H) Spindle shortening during the prolonged metaphase of *Int6* RNAi cells. The broken red line indicates the time of metaphase plate formation; the error bars indicate SEM.

Previous studies showed that Int6-depleted S2 cells exhibit short spindles and are delayed in metaphase [22]. To further define the mitotic phenotype elicited by Int6 depletion we re-examined S2 cells treated for 5 days with *Int6* dsRNA, a treatment that resulted in a drastic reduction of Int6 (Fig 1A). We chose a 5-day RNAi treatment because at 4 days Int6 was not sufficiently depleted; we only examined dividing cells with a minimal karyotype (~ 12 chromosomes;[28]). Thus, we limited our observations to cells that were unlikely to carry mitotic defects generated by reduction of Int6 during the previous cell cycles. Staining for both tubulin and DNA revealed that most dividing *Int6* RNAi cells are arrested in metaphase and exhibit short and compact spindles (Fig 1B–1D). Notably, approximately 70% of these metaphases displayed a tight chromosome alignment comparable to that observed in live metaphases just before anaphase. Anaphase and telophase figures of Int6-depleted cells were also shorter than their normal counterparts, but did not exhibit gross defects in chromosome segregation (S1 Fig).

We also examined cell division in live *Int6* RNAi cells that express mCherry-tubulin and histone-GFP. Here again, we limited our observation to cells with a minimal karyotype. We time-lapse recorded mitosis of these RNAi cells starting from prometaphase; they remained in metaphase for much longer times (up to 3 hours) than control cells, which entered anaphase within 35 min after metaphase plate formation (Fig 1E and 1F; S1 and S2 Movies). Interestingly, early prometaphase spindles of control and *Int6* RNAi cells were similar in length, but approximately 10 minutes before formation of the metaphase plate the spindles of Int6-depleted cells started to shorten, and after 30 minutes spent in metaphase they were 35% shorter than those of untreated controls (Fig 1H). We also video-recorded chromosome movement during anaphase; we found that the chromosomes of control cells (n = 12) and *Int6* RNAi cells (n = 16) move at 1.10 ± 0.09 and 0.35 ± 0.04 μm/min, respectively (Fig 1E and 1G). Thus, Int6 deficiency slows down chromosome movement during anaphase A.

### Int6 depletion results in centromere/kinetochore deformation, low intra-KD and high inter-KD

**Fig 2:**
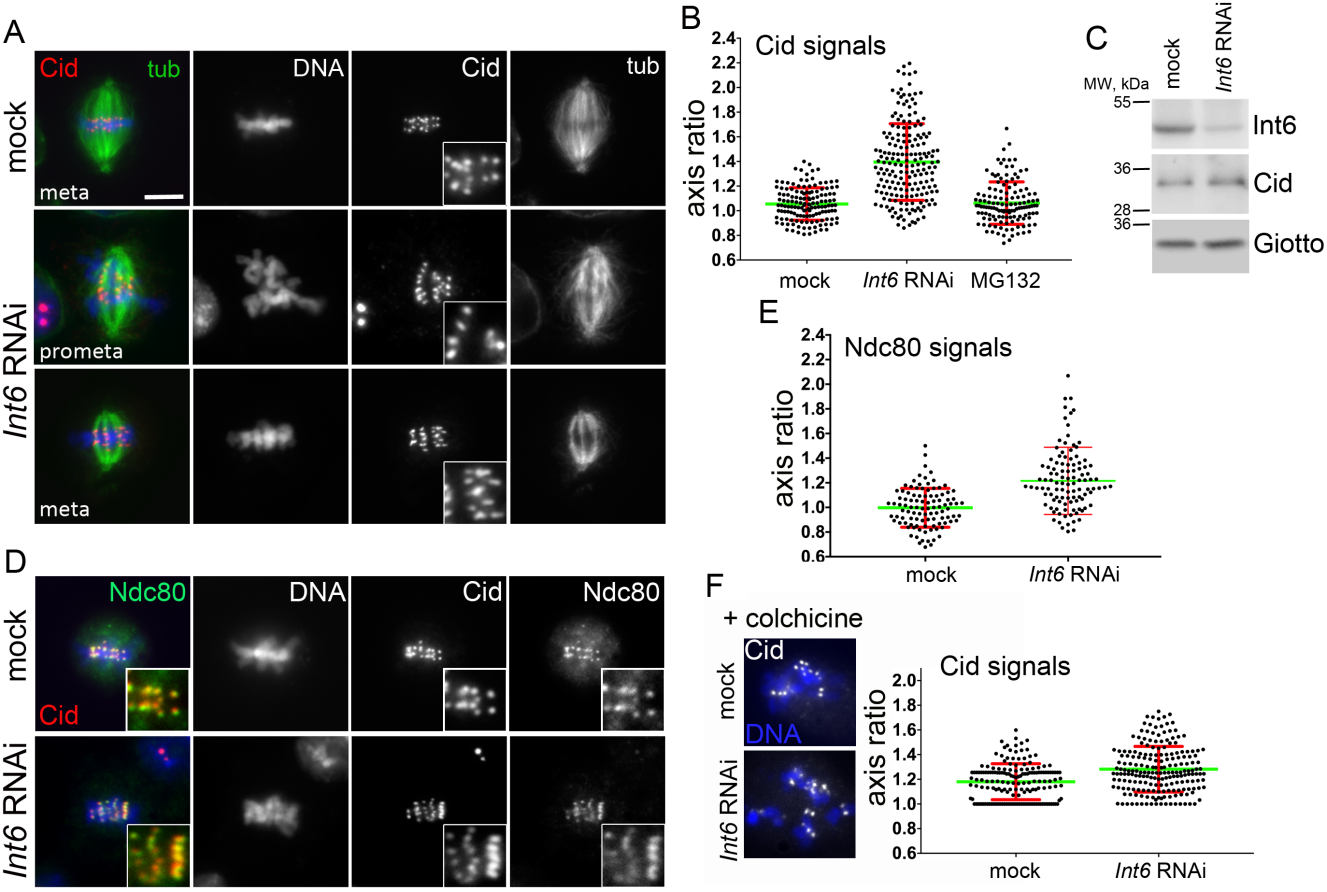
Int6 depletion leads to centromere/kinetochore deformation. (A) Metaphases (meta) and prometaphases (prometa) from mock-treated and *Int6* RNAi cells stained for DNA (blue), tubulin and Cid. In Int6-depleted metaphases (see insets), many Cid signals are elongated and placed perpendicularly to the spindle axis Scale bar, 5 μm. (B) Measurement of the long/short axis ratios of Cid signals in metaphases from mock-treated control cells, *Int6* RNAi cells and cells treated for 6 hours with the proteasome inhibitor MG132. The average ratio (green line; red lines indicate SD) observed in Int6-depleted cells is significantly higher than that seen in either control or MG132-treated cells (p< 0.0001 in the Student's t test). The average ratios of control and MG132-treated cells are not significantly different. (C) Western blotting showing that *Int6* RNAi and mock RNAi cells exhibit similar Cid levels; Giotto was used as a loading control. (D) Metaphases from mock-treated and *Int6* RNAi cells co-stained for DNA (blue), Cid and Ndc80. (E) In Int6-depleted cells, the average long/short axis ratio of the Ndc80 signals is significantly higher than in controls (p< 0.0001; Student's t test). (F) In colchicine-treated metaphases the average long/short axis ratio of Cid signals observed in Int6-depleted cells is significantly higher than in mock treated cells (p < 0.001; Student's t test).

Immunostaining for Cid (the *Drosophila* CenpA homolog) revealed that in metaphases of Int6-depleted cells many Cid signals are abnormally shaped compared to those of control cells. These signals were ellipsoid or cylindrical in shape and had their major axis oriented orthogonally with respect to the longitudinal spindle axis (Fig 2A). To quantify the effect of Int6 depletion on Cid signals we focused on those that were distinct from other signals and measured the ratio between their major and minor axis. For all signals we considered as major axis the one orthogonal to the spindle axis (see Materials and methods for details). We found that in Int6-depleted metaphases these ratios were significantly higher than in controls, suggesting that loss of Int6 leads to a deformation of metaphase centromeres (Fig 2B). We next asked whether the centromere deformation reflected an increase in the Cid amount (possibly due to its reduced degradation; see below). Western blotting showed *Int6* RNAi and mock RNAi cells exhibit very similar Cid levels (Fig 2C), suggesting that the centromeres of Int6-depeleted cells are morphologically abnormal and not simply larger than those of control cells.

We also co-stained *Int6* RNAi metaphases for both Cid and the outer kinetochore component Ndc80 [29]. We found a high degree of coincidence between the two fluorescent signals (Fig 2D). Consistent with this observation, in Int6-depleted metaphases the ratios between the major and minor axes of the Ndc80 signals were significantly higher than in controls (Fig 2E). Thus, in Int6-depleted cells both the centromere chromatin and the outer kinetochore are similarly deformed.

To ascertain whether the kinetochore deformation phenotypes observed in *Int6* RNAi cells was due to their prolonged arrest in metaphase, we examined cells treated with the proteasome inhibitor MG132, which is known to block S2 cells in metaphase [30]. Cells were treated with MG132 for 6 hours, fixed and then stained for tubulin, Cid and DNA. As expected, in MG132 treated cultures all dividing cells were blocked in metaphase with well-aligned chromosomes and morphologically normal centromeres (Fig 2B). Thus, the centromere phenotype seen in *Int6* RNAi cells cannot be a direct consequence of the metaphase arrest suffered by these cells.

We next examined Cid-stained centromere regions in Int6-depleted and control cells incubated for 2 hours with colchicine (Fig 2F). To measure the shape of these regions we used the fit-ellipse function of the ImageJ software, which provides the length of the major and minor axis of the fluorescent signal (see Materials and methods for details). In colchicine-treated Int6-depleted cells, the average axial ratio of Cid signals was higher than in colchicinized controls, but the difference in ratios was reduced compared to that observed in non-colchicinized cells (compare Fig 2B and 2F). These results indicate that the presence of the spindle MTs contributes to centromere/kinetochore deformation in Int6-deficient cells.

**Fig 3:**
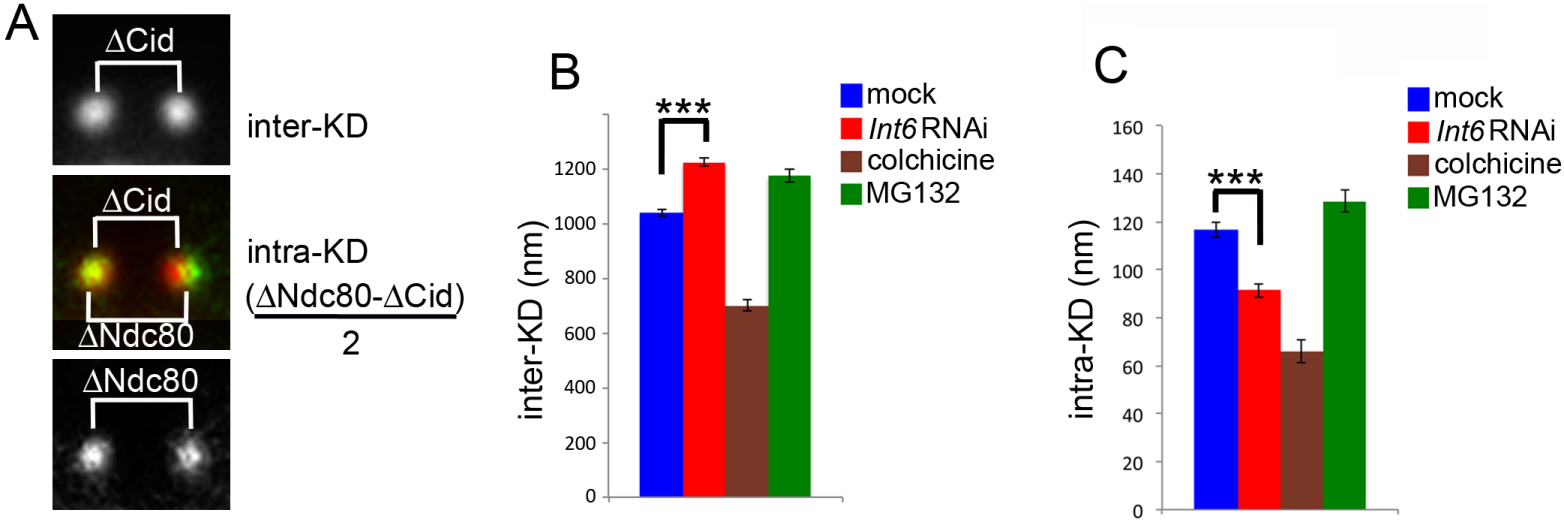
Int6 depletion affects both intra-KD and inter-KD. (A) Method used to measure the intra-kinetochore (intra-KD) and inter-kinetochore (inter-KD) distances. ΔNdc80 and ΔCid are the distances between the centers of the Ndc80 and the Cid signals. (B, C) Inter-KDs (± SEM) (B) and intra-KDs (± SEM) (C) observed in Int6-depleted cells (194 Cid/Ndc80 signal pairs), mock-treated cells (control; 182 Cid/Ndc80 signal pairs) colchicine-treated cells (negative control, 142 Cid/Ndc80 signal pairs) and MG132-treated cells (positive control, 160 Cid/Ndc80 signal pairs). ***, significantly different in the Student’s t test with p < 0.0001.

Although the Int6-deficient metaphases show tightly aligned chromosomes, they exhibit a strong delay in anaphase entry, suggesting that the SAC is not satisfied. In *Drosophila*, satisfaction of the SAC requires axial stretching of the kinetochores that is manifested as an increase in the intra-kinetochore distance (intra-KD) [31]; namely, the distance between the outer corona marked by proteins such as Ndc80 and the inner kinetochore marked by Cid/CenpA (Fig 3A). SAC is satisfied when the intra-KD is elevated, while it remains active when the intra-KD is relatively low. In contrast, the inter-kinetochore distance (inter-KD; the distance between the Cid/CenpA signals associated with sister chromatids) does not affect the SAC activity [31]. To assess the inter- and intra-KDs we examined Int6-depleted and control metaphases stained for both Cid and Ndc80; metaphases from colchicine-treated cells or cells treated with the proteasome inhibitor MG132 served as negative and positive controls for intra-KD, respectively [32–34]. In *Int6* RNAi metaphases, the inter-KD was significantly higher than the control value but the intra-KD was significantly lower than that seen in control metaphases (Fig 3B and 3C). The relatively high inter-KD indicates that the spindle MTs exert tension on the bioriented sister kinetochores leading to the formation of a compact metaphase plate with tightly aligned chromosomes. However, the low intra-KD of these chromosomes is likely to prevent SAC silencing and anaphase onset [31].

**Fig 4:**
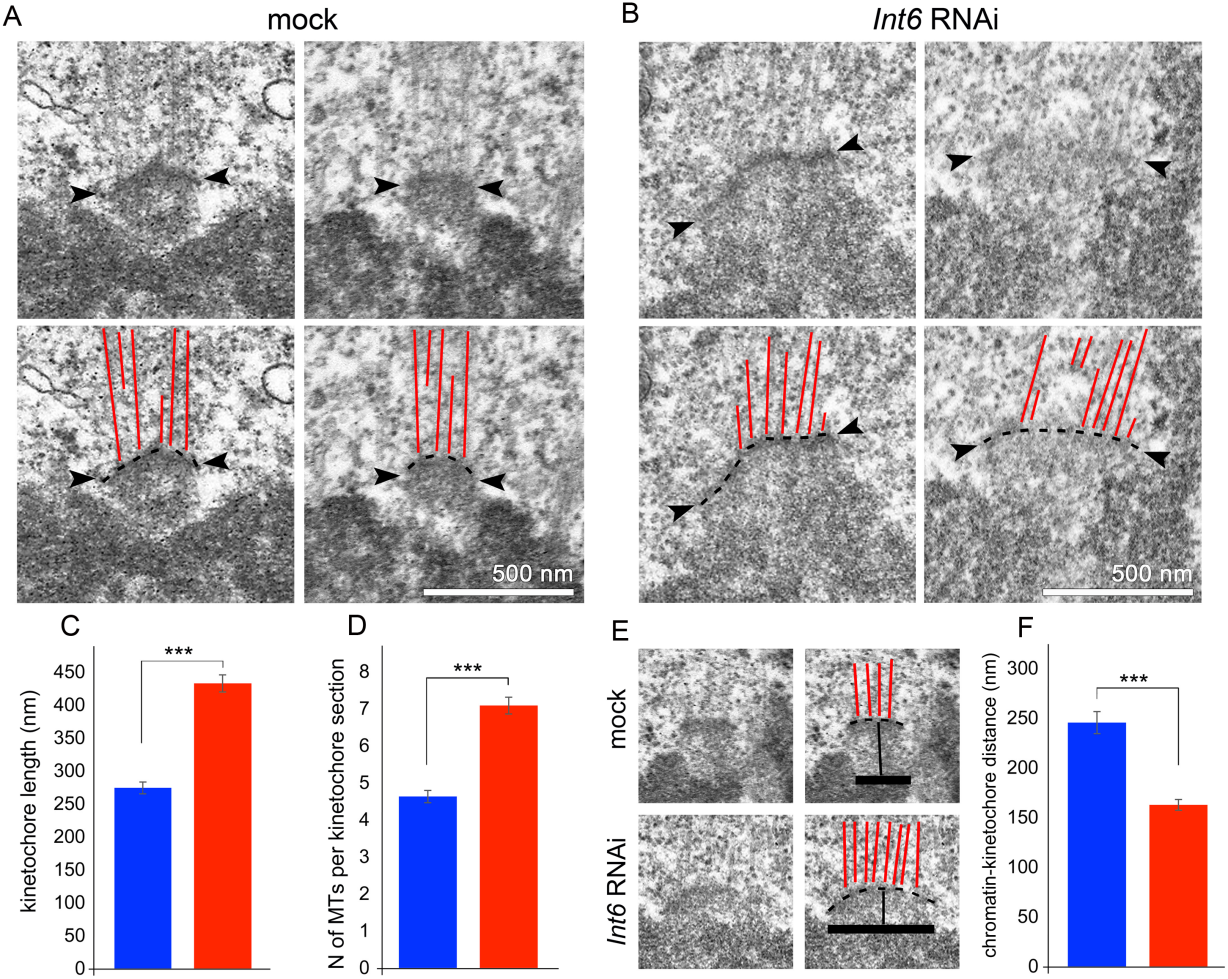
TEM analysis of *Int6* RNAi metaphases detects kinetochore stretching and reduced distance between chromatin and the kinetochore plate. (A, B) Kinetochores of mock-treated controls (A) and *Int6* RNAi cells (B) visualized by TEM in single ultrathin sections. Arrowheads and dotted lines denote the outer kinetochore plate. (C) Average length (± SEM) of the kinetochore plate in controls and *Int6* RNAi treated cells. (D) Average number of MTs (± SEM) emanating from the kinetochore in controls and *Int6* RNAi cells. (E) Examples of kinetochores showing that the distance between the chromatin and kinetochore plate in *Int6* RNAi cells is higher than in controls; the broken lines and the thick continuous lines indicate the outer edges of the chromatin and the kinetochore plates, respectively. (F) Average chromatin-outer kinetochore plate distance (± SEM) in control and *Int6* RNAi cells. ***, significantly different in the Student’s t test with p < 0.0001.

To obtain additional insight into the kinetochore structure of Int6-depleted cells, we performed a transmission electron microscopy (TEM) analysis by examining single ultrathin sections (of approximately 30 nm) of kinetochores displaying end-on attached MTs (Fig 4A and 4B). In control cells, the mean length of kinetochore plates (24 metaphases, 66 kinetochores) was 279 ± 9 nm, while the mean number of MTs emanating from the kinetochores was 4.6 ± 0.1 (Fig 4C and 4D). In Int6-depleted cells (25 metaphases, 68 kinetochores), both the mean kinetochore length (433 ± 13 nm) and MT number (7.1 ± 0.2) were significantly higher than in controls (Fig 4C and 4D). Interestingly, the kinetochore length (433/279 = 1.55) and the MT number (7.1/4.6 = 1.54) ratios between *Int6* RNAi and control cells are virtually identical, indicating that the MT capturing ability of deformed kinetochores is the same as that of normal kinetochores. It should be noted that the single ultrathin sections we examined contain only a fraction of the MTs that are normally attached to a *Drosophila* kinetochore. Analyses of 100-nm-thick serial sections have previously suggested that S2 cell kinetochores are associated with an average of 11 MTs [35]. We examined cross-sections through 5 kinetochores of control cells and found 10–15 end-on attached MTs, consistent with previous results.

TEM analysis also revealed a 50% decrease in the mean distance between the edge of chromatin and the outer edge of the kinetochore plate in metaphases of Int6-depleted cells. (Fig 4E and 4F; 22 kinetochores in 14 cells for controls; 30 kinetochores in 13 cells for *Int6* RNAi cells). This decrease is consistent with the short intra-kinetochore distance between the inner (Cid) and outer (Ndc80) kinetochore components observed by light microscopy (Fig 3C).

### Factors underlying the mitotic phenotype elicited by Int6 depletion

**Fig 5:**
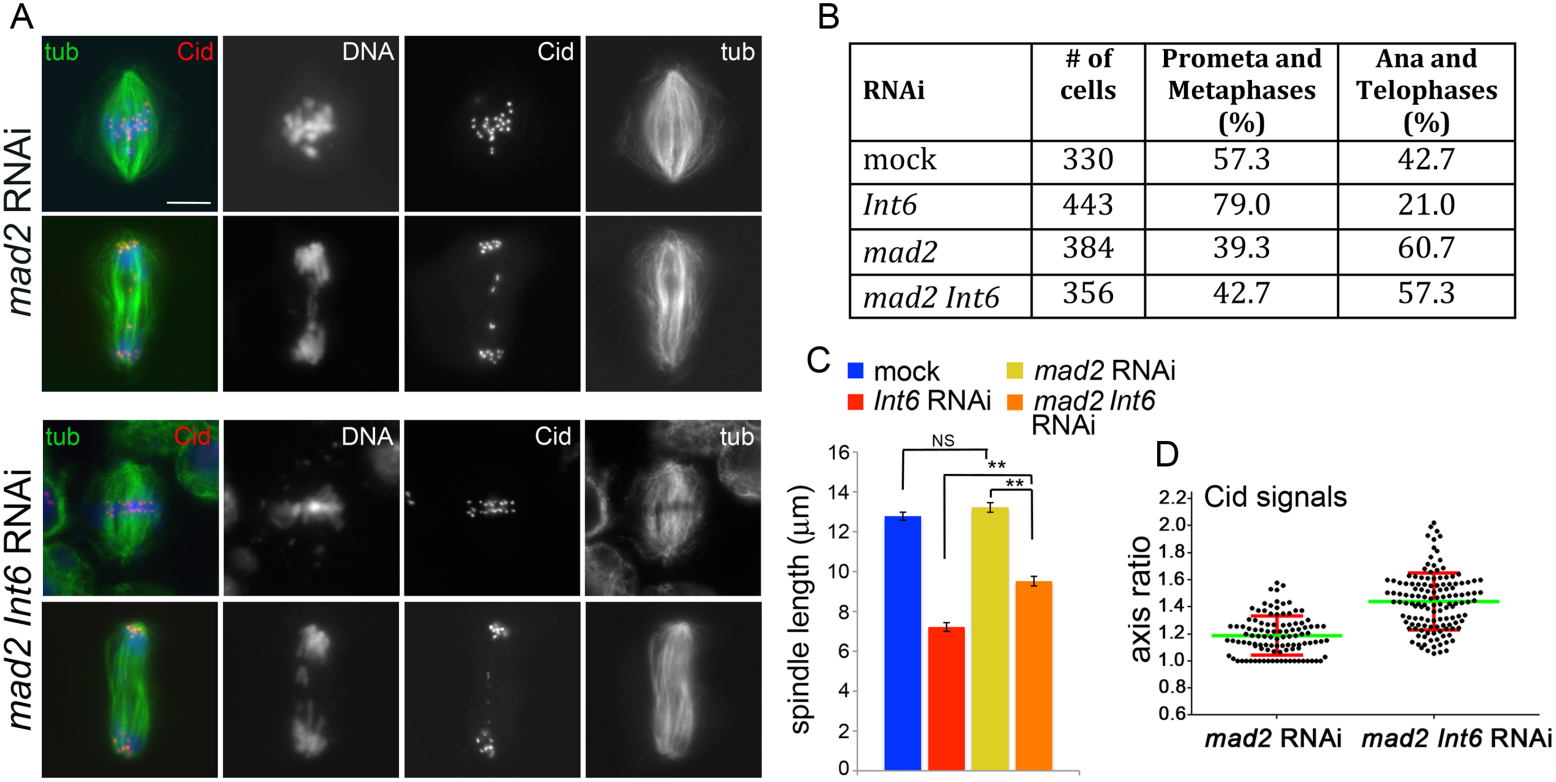
Down-regulation of mad2 partially rescues the mitotic defects caused by Int6 deficiency. (A) Metaphases and anaphases observed in *mad2* RNAi cells and in *mad2 Int6* double RNAi cells stained for tubulin (tub), Cid and DNA (blue). Scale bar, 5 μm. (B) Frequencies of mitotic figures observed in mock treated cells and in RNAi cells for the indicated genes; prometaphases (prometa) and metaphases have been pooled. The frequencies of anaphases (ana) plus telophases seen in *mad2* RNAi and *Int6 mad2* RNAi cells are not significantly different in the χ^2^ test. (C) Average metaphase spindle length (± SEM) observed in mock treated and RNAi cells for the indicated genes (80 metaphase spindles measured in each cell type). **, significantly different in the Student’s t test with p < 0.001; NS not significantly different. (D) The average long/short axis ratio of Cid signals observed in metaphases of *mad2 Int6* double RNAi cells is significantly higher than that found in *mad2* RNAi metaphases (p< 0.0001; Student's t test).

**Fig 6:**
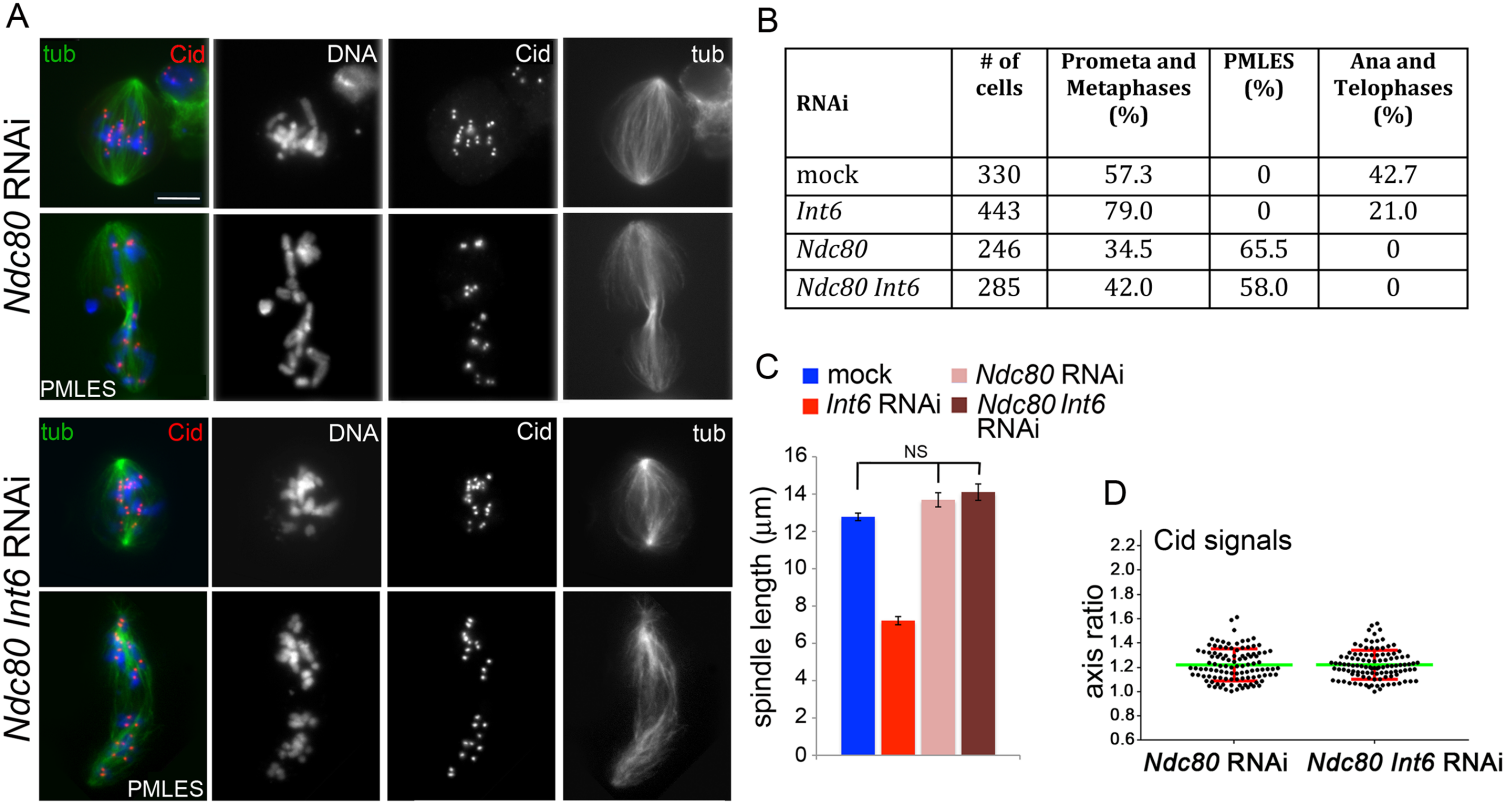
Ndc80 deficiency is epistatic over Int6 deficiency. (A) Prometaphase-like figures and prometaphase like cells with elongated spindles (PMLES; see text for precise definition) observed in *Ndc80* RNAi cells and in *Ndc80 Int6* double RNAi cells stained for tubulin (tub), Cid and DNA (blue). (B) Frequencies of mitotic figures observed in mock-treated cells and in RNAi cells for the indicated genes. The frequencies of PMLES observed in double RNAi cells and in cells depleted of Ndc80 only are not significantly different in the χ^2^ test. (C) Average metaphase spindle length (± SEM) observed in mock treated and RNAi cells for the indicated genes (80 metaphase spindles measured in each cell type). NS, not significantly different in the Student's t test. (D) The long/short axis average ratio of Cid signals in metaphases from *Ndc80 Int6* double RNAi cells is not significantly different (Student's t test) from that of metaphases from *Ndc80* RNAi cells.

To obtain further insight into the mechanisms underlying the mitotic phenotype caused by loss of Int6 we performed double RNAi experiments. We first carried out RNAi against *Int6* and *mad2*, which encodes a component of the SAC machinery that mediates metaphase arrest [36]. Double RNAi cells for *mad2* and *Int6* displayed a relief from the partial metaphase arrest observed in *Int6* RNAi cells, showing an anaphase frequency comparable to that found in cells depleted of Mad2 only (Fig 5A and 5B). These cells also showed metaphase spindles that were significantly longer than those of cell depleted of Int6 only, but still significantly shorter than control spindles (Fig 5C). Finally, while in *mad2* RNAi cells the centromere shape was normal, in *mad2 Int6* double RNAi cells the centromeres were deformed showing an average axial ratio comparable to that observed in Int6-depleted cells (Fig 5D; see also Fig 2B; see Materials and methods for the procedure used for Cid signal measurement). These findings suggest that the metaphase arrest phenotype caused by Int6 depletion depends on a persistent SAC activity, and also demonstrates that prolongation of mitosis is not required for centromere deformation in Int6-depleted cells. Because Int6-depleted cells exhibit a limited centromere deformation after colchicine treatment, we sought to confirm the role of spindle MTs in the genesis of this phenotype. We thus performed double RNAi against *Int6* and *Ndc80*, which encodes a protein that mediates MT-kinetochore attachment and is required for proper SAC activity [29,37]. Double RNAi cells showed the same phenotype as cells depleted of Ndc80 only; namely, they displayed morphologically normal centromeres/kinetochores, metaphase spindles of regular length, and scattered chromosomes (Fig 6A–6D). *Ndc80* RNAi cells and double RNAi cells also showed many cells with elongated spindles associated with chromosomes with unseparated sister chromatids (Fig 6A and 6B). These peculiar cells, show high levels of Cyclin B, suggesting that they are metabolically in metaphase [22]. Similar mitotic figures have been previously observed in Cid (the *Drosophila* homologue of CenpA)-depleted cells and have been named pseudo ana-telophases (PATs, [22]) because they often show central spindle-like structures associated with irregular contractile rings. Here, to avoid possible confusion, we designate them as prometaphase-like cells with elongated spindles (PMLES).

The observations on *mad2 Int6* and *Int6 Ndc80* double RNAi cells indicate that the short spindle phenotype depends on both kinetochore-MT attachment and SAC activity. They also suggest that the centromere deformation phenotype depends on kinetochore-MT attachment but not on SAC activity. This conclusion is consistent with the observation that colchicine treated Int6-depleted cells exhibit fewer misshapen centromeres when compared to colchicine-treated controls. It is indeed likely that at the time of colchicine treatment a fraction of Int6-deficient cells was in metaphase and had already experienced kinetochore-MT interaction and undergone centromere deformation.

### Int6 depletion decreases the rate of MT poleward flux

Shortening of the spindle suggests a change in the dynamic of spindle microtubules in Int6-depleted cells. To reveal the nature of this change we measured the rate of microtubule flux and microtubule turnover both within the K-fibers and near the spindle poles.

**Fig 7:**
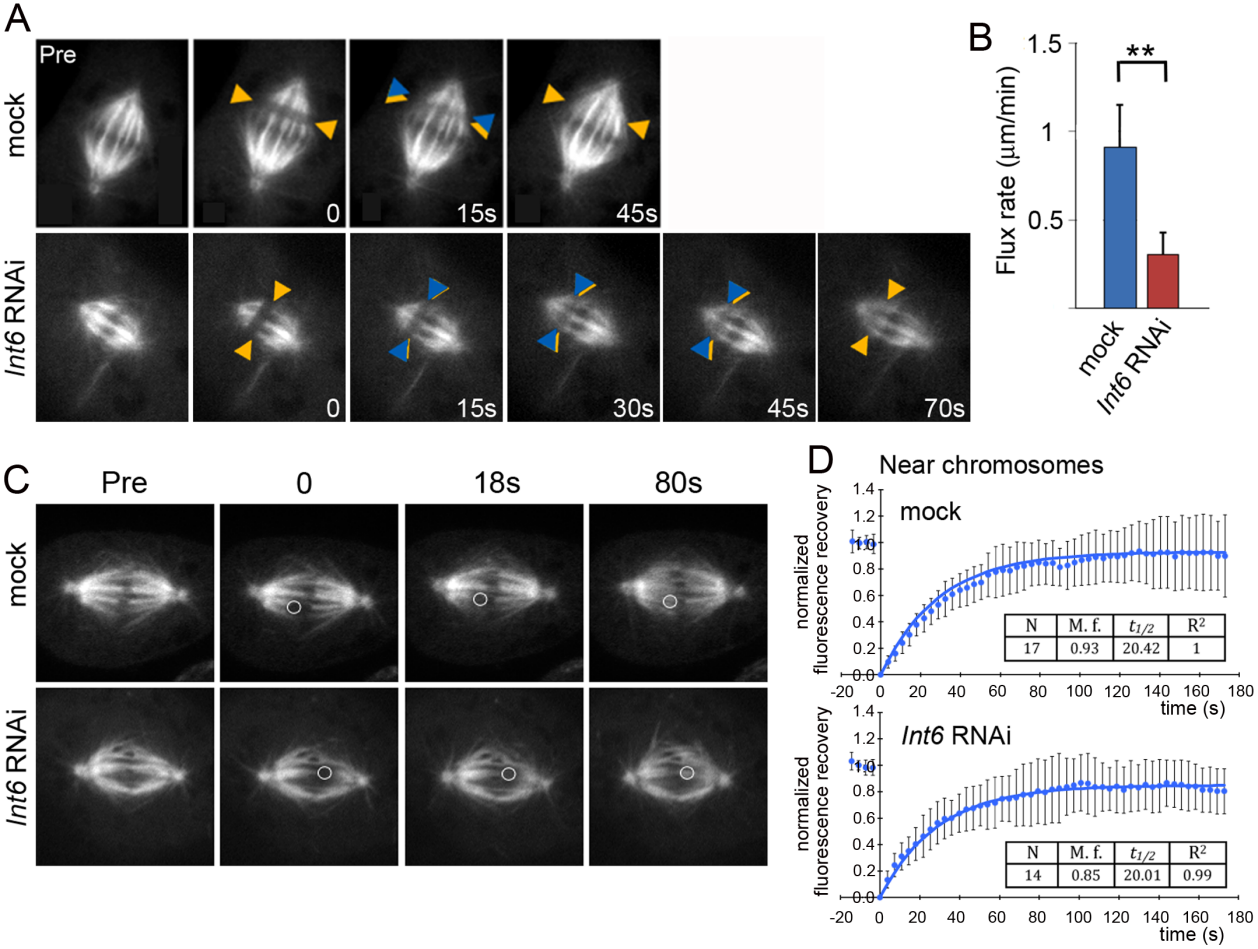
Int6 depletion reduces the rate of MT flux but does not affect microtubule turnover within K-fibers. (A) Visualization of MT flux rates in mock-treated and *Int6* RNAi cells. Orange and blue arrowheads mark the initial and current positions of bleached regions, respectively. Pre, prebleached spindle. Numbers refer to seconds after initial photobleaching. (B) Poleward flux rates (± SD) in control and *Int6* RNAi cells. **, significantly different in the Student’s t test with p < 0.001. (C-D) FRAP analysis of individual K-fibers near kinetochores. Bleached areas are denoted by circles. (C) Averaged curves and recovery parameters (D).

The MT poleward flux is the continuous flow of the MT subunits towards the spindle poles driven by tubulin addition at their plus ends and tubulin disassembly at their minus ends [23,38]. Depletion of Int6 resulted in a significant decrease in the poleward velocity of photobleached marks during metaphase: from 0.91 ± 0.24 μm/min in control cells (n = 20) to 0.30 ± 0.12 in Int6-depleted cells, (n = 17) (Fig 7A and 7B). The reduction in flux rate upon *Int6* RNAi is consistent with the slow movement of chromosomes during anaphase observed in these cells. Because poleward flux involves continuous polymerization of MTs at the kinetochores and balanced depolymerization at the spindle poles, spindle shortening observed in Int6-depleted cells strongly suggests that incorporation of new subunits into the plus ends of kinetochore-attached MTs occurs at a lower rate compared to control.

In contrast, all parameters of Fluorescence Recovery After Photobleaching (FRAP) within K-fibers near the kinetochores were similar in Int6-depleted and control cells (Fig 7C and 7D). This suggests that detachment and reattachment of K-fiber microtubules to the kinetochore are not affected by Int6 depletion. Control and Int6-depleted cells also showed comparable FRAP parameters when photobleaching was performed near the spindle poles (S2 Fig).

### The phenotype caused by Int6 depletion is due to defective Klp67A degradation

Int6 has been implicated in the ubiquitin-mediated protein degradation pathway [14,16] and may therefore mediate proteolysis of factors that regulate MT behavior. We reasoned that loss of Int6 could result in an accumulation of a protein that would reduce net growth of the MT plus ends embedded in the kinetochore. A good candidate for this role was the plus end-directed Klp67A kinesin-like protein, which localizes at kinetochores and represses MT plus end growth [23–26]. We thus envisaged that loss of Int6 could result in failure of Klp67A degradation and that the consequent accumulation of this protein could affect MT behavior in an opposite fashion to Klp67A depletion.

**Fig 8:**
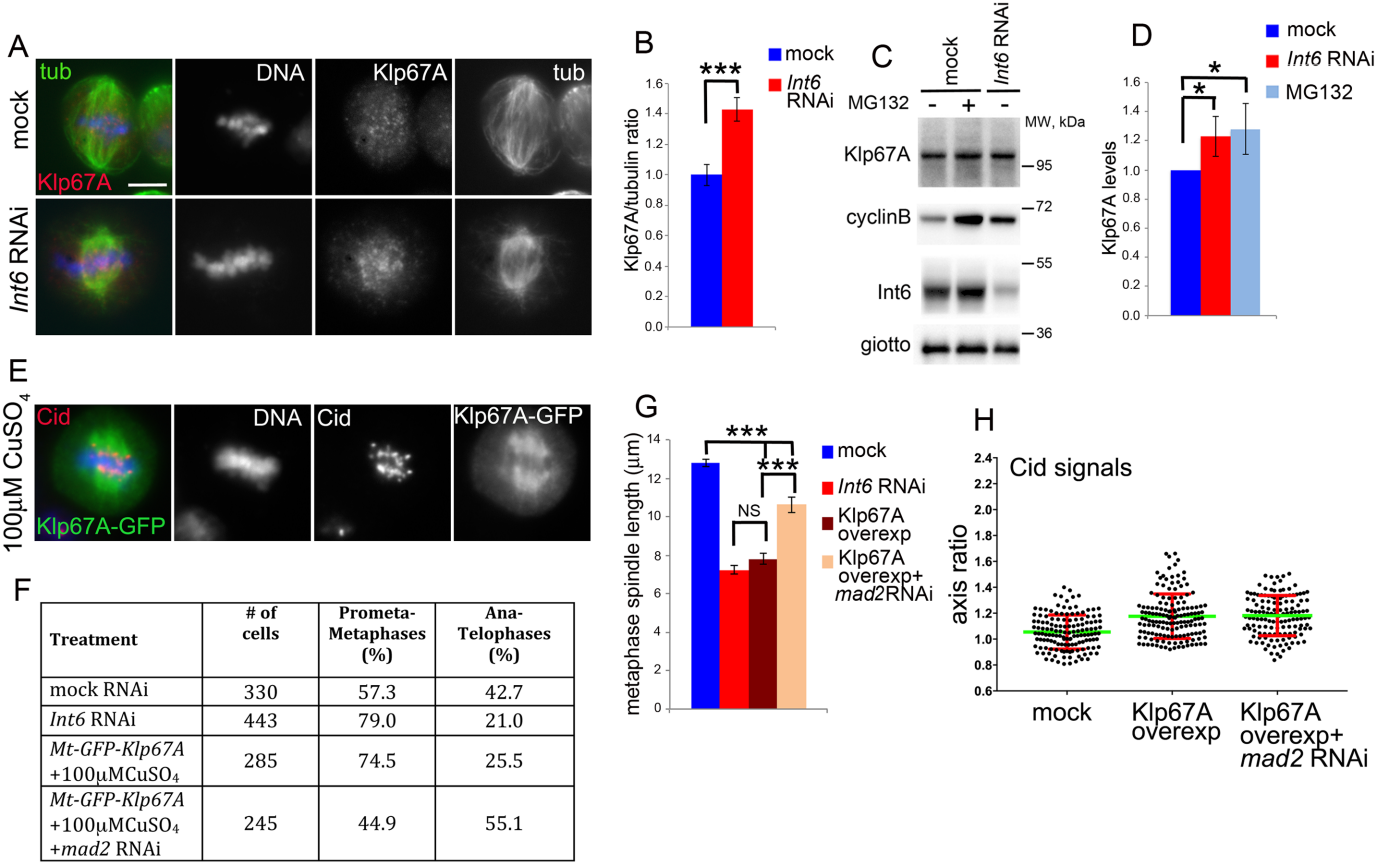
The mitotic phenotype elicited by Int6 deficiency is caused by Klp67A accumulation near kinetochores. (A) Klp67A immunostaining shows that Int6-depleted cells accumulate endogenous Klp67A at kinetochores and in the central part of metaphase spindles. (B) Average ratios between spindle/kinetochore-associated Klp67A and tubulin fluorescence (± SEM) in control (n = 40) and Int6-depleted (n = 40) cells. ***, significantly different in the Student’s t test with p < 0.0001. (C, D) Western Blots with extracts from control and Int6-depleted cells; Cyclin B was used as a positive control for MG132 treatment (C), and quantitation from 4 independent experiments of the Klp67A band intensity (± SEM) relative to the Giotto band (D); * significantly different in the Student’s t test with p < 0.05. (E) Accumulation of overexpressed Klp67A-GFP along the entire spindle. (F) Frequencies of mitotic figures in mock-treated, *Int6* RNAi and Klp67A-GFP-overexpressing cells. The frequency of ana-telophases in Klp67A-GFP overexpressing cells is lower (p < 0.001; χ^2^ test) than in controls but is not significantly different from that of *Int6* RNAi cells. RNAi against *mad2* in Klp67A-GFP overexpressing cells significantly increases the frequency of ana-telophases compared to Klp67A-GFP overexpressing cells not exposed to *mad2* dsRNA (p < 0.001; χ^2^ test) (G) Average metaphase spindle length (± SEM) observed in mock-treated cells, *Int6* RNAi cells, and Klp67A-GFP overexpressing cells either exposed or not exposed to RNAi against *mad2* (80 metaphase spindles measured in each cell type). ***, significantly different in the Student’s t test with p < 0.0001; NS, not significantly different. (H) The average long/short axis ratios of Cid signals in metaphases of Klp67A-GFP overexpressing cells, treated or not treated with *mad2* dsRNA, are significantly higher than that of control cells (p < 0.001; Student’s t test).

To test this possibility, we performed immunostaining experiments to determine whether Int6-depleted cells accumulate Klp67A. In control cells, Klp67A was associated with the spindle MTs and accumulated on the kinetochores. In *Int6* RNAi cells, Klp67A displayed an accumulation on both the kinetochores and the spindle MTs. Measurements of fluorescence intensity revealed that in Int6-depleted metaphases there is a significant increase in spindle- and kinetochore- associated Klp67A compared to controls (Fig 8A and 8B). A small but significant increase in Klp67A was also detected by Western blotting (Fig 8C and 8D). Given that the mitotic index in S2 cells is 3%, the limited Klp67A increase observed in Western blots is consistent with the hypothesis that Klp67A undergoes ubiquitin/proteasome-mediated degradation mainly during mitosis.

We next asked whether overexpression of Klp67A could mimic the phenotype elicited by Int6 depletion. We overexpressed *Klp67A-GFP* by placing the gene under the control of the *Drosophila* metallothionein (Mt) inducible promoter. After addition of copper sulfate (100 μM CuSO_4_) to the culture medium, dividing cells overexpressing Klp67A-GFP were easily recognizable for their fluorescent spindles (Fig 8E). Fixed cells expressing Klp67A-GFP displayed different degrees of fluorescence; to define their mitotic phenotype we examined only cells that were in the top 20% for fluorescence intensity. An analysis of these cells revealed that they have short spindles and are delayed in their progression through metaphase just like Int6-depleted cells (Fig 8F and 8G). In addition, they displayed a significant increase in the long/short axis ratio of the Cid (CenpA) signals compared to controls (Fig 8H), but this increase was lower than that observed in Int6-depleted cells (see Fig 2). RNAi against *mad2* in Klp67A-GFP overexpressing cells rescued the metaphase arrest phenotype, led to a partial recovery of spindle length but did not rescue centromere deformation (Fig 8F–8H). Thus, the defects caused by Klp67A-GFP overexpression are similar to those caused by Int6 depletion.

To extend the comparison between Int6-depleted and Klp67A-GFP overexpressing cells, we generated a cell line constitutively expressing mCherry-tubulin and carrying *Mt-Klp67A-GFP*. Here again, we induced Klp67A-GFP expression by addition of 100 μM of CuSO_4_ and focused on spindles that appeared to be in the top 30% as to green fluorescence. In the cells examined, chromosomes were not marked but metaphases were recognizable because they displayed at dark stripe at the spindle equator, in correspondence of the congressed chromosomes. We analyzed spindle behavior by filming newly formed bipolar spindles from prometaphase to metaphase; we found that they exhibit a considerable shortening as they proceed to and through metaphase, just like those of Int6-depleted cells (S3 Fig). In addition, we measured FRAP of kinetochore-associated mCherry-marked MT bundles in metaphases overexpressing Klp67A-GFP (green spindles); non-green metaphases from the same cultures were used as controls. We found that Klp67A-GFP overexpression does not alter the fluorescence recovery time compared to cells expressing normal Klp67A levels (S4 Fig). Thus, also the *in vivo* analysis indicates Klp67A-GFP overexpressing cells and Int6-depleted cells exhibit similarities in their mitotic behavior.

**Fig 9:**
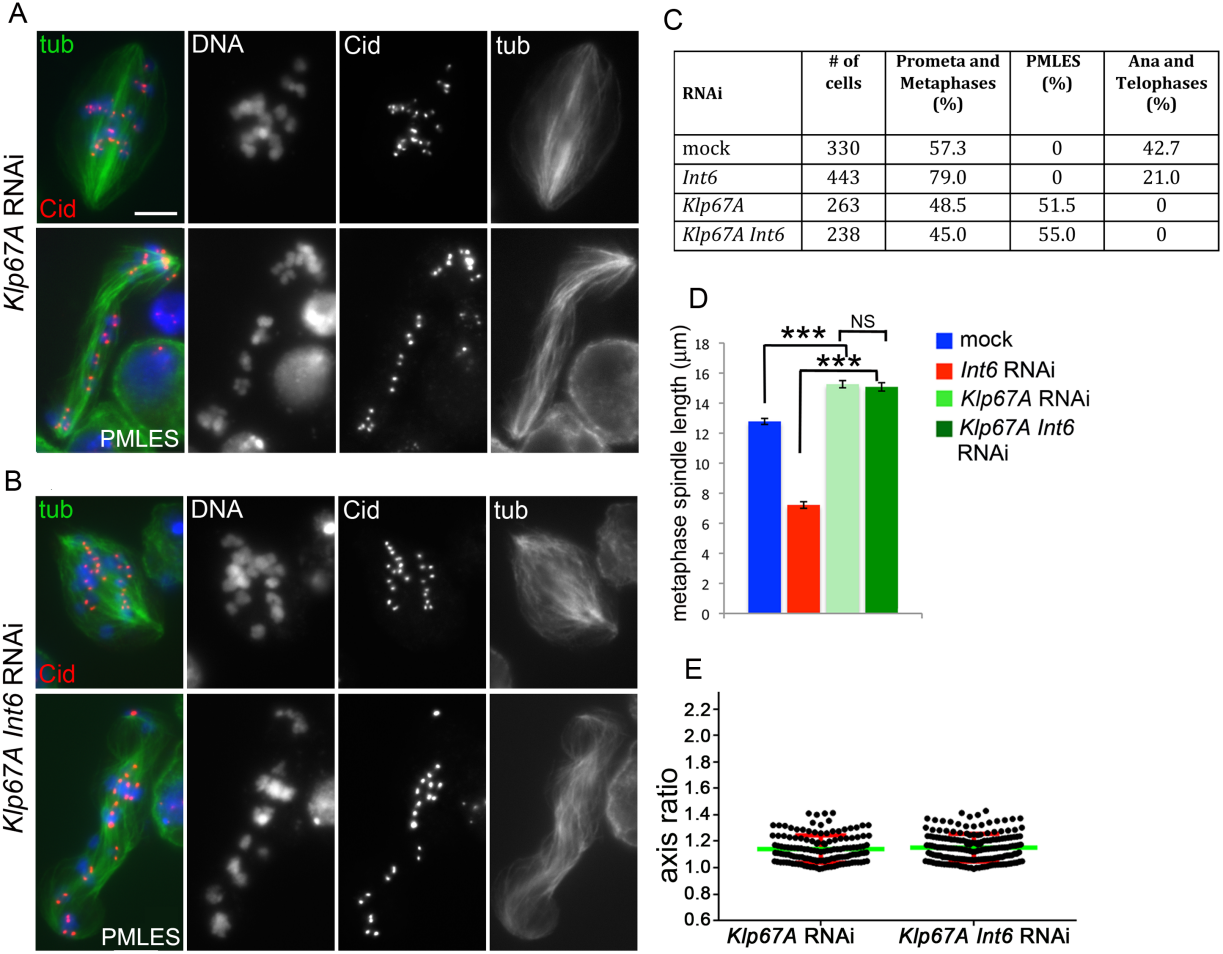
Klp67A deficiency is epistatic over Int6 deficiency. (A, B) Prometaphase-like figures and PMLES observed in *Klp67A* RNAi (A), and *Klp67A Int6* double RNAi cells (B) stained for tubulin (tub), Cid and DNA (blue). (C) Frequencies of mitotic figures observed in mock-treated cells and RNAi cells against the indicated genes. The frequencies of PMLES observed in double RNAi cells and in cells depleted of Klp67A only are not significantly different in the χ^2^ test. (D) Average metaphase spindle length (± SEM) observed in mock-treated cells and RNAi cells against the indicated genes (80 metaphase spindles measured in each cell type). ***, significantly different in the Student’s t test with p < 0.0001; NS, not significantly different. (E) The average long/short axis ratios of Cid signals in metaphases from *Klp67A* RNAi cells and *Klp67A Int6* double RNAi cells are not significantly different in the Student's t test.

These results suggest that the mitotic defects caused by Int6 depletion are largely due to an accumulation of Klp67A at kinetochores. To test this hypothesis we compared *Int6* and *Klp67A* double RNAi cells with cells lacking Klp67A only. We observed identical phenotypes with both cell samples showing normal kinetochores, misaligned metaphase chromosomes and spindles longer than those of mock-treated cells (Fig 9A–9E). In addition, both cells samples did not show anaphases and displayed only PMLES (Fig 9A–9C), suggesting that they are both defective in kinetochore-MT interaction [22].

We also compared *Int6* and *Klp10A* double RNAi cells with cells lacking *Klp10A* only. We performed this experiment because Klp10A is known to destabilize MT minus ends at the spindle poles [26,39]. We found that in *Klp10* RNAi cells the spindles are substantially longer that in control cells, consistent with previous results [26,39] (S5 Fig). Cells deficient for both Int6 and Klp10 displayed spindles significantly longer than those of Int6-depleted cells but still shorter than control spindles (S5 Fig). These results indicate that Int6 and Klp10A play antagonistic roles during spindle assembly, and suggest that Int6 deficiency affects MT plus ends at kinetochores, consistent with the flux and FRAP results (see Fig 7).

We finally investigated whether the subcellular localization of Int6 correlates with Klp67A localization. We generated an anti-Int6 antibody, which specifically recognized the Int6 protein in Western blots (Figs 1A and S6). Immunostaining of S2 cells with this antibody revealed that Int6 is uniformly distributed in both interphase and mitotic cells; the staining was strongly reduced in *Int6* RNAi cells, demonstrating the antibody specificity (S6 Fig). The diffuse Int6 distribution does not conform to Klp67 localization at kinetochores and spindle MTs. This suggests that the role of Int6 is not restricted to a specific cellular compartment, consistent with its association with different protein complexes involved in diverse functions [12–14].

### Int6-depleted cells accumulate non-ubiquitinated Klp67A

**Fig 10:**
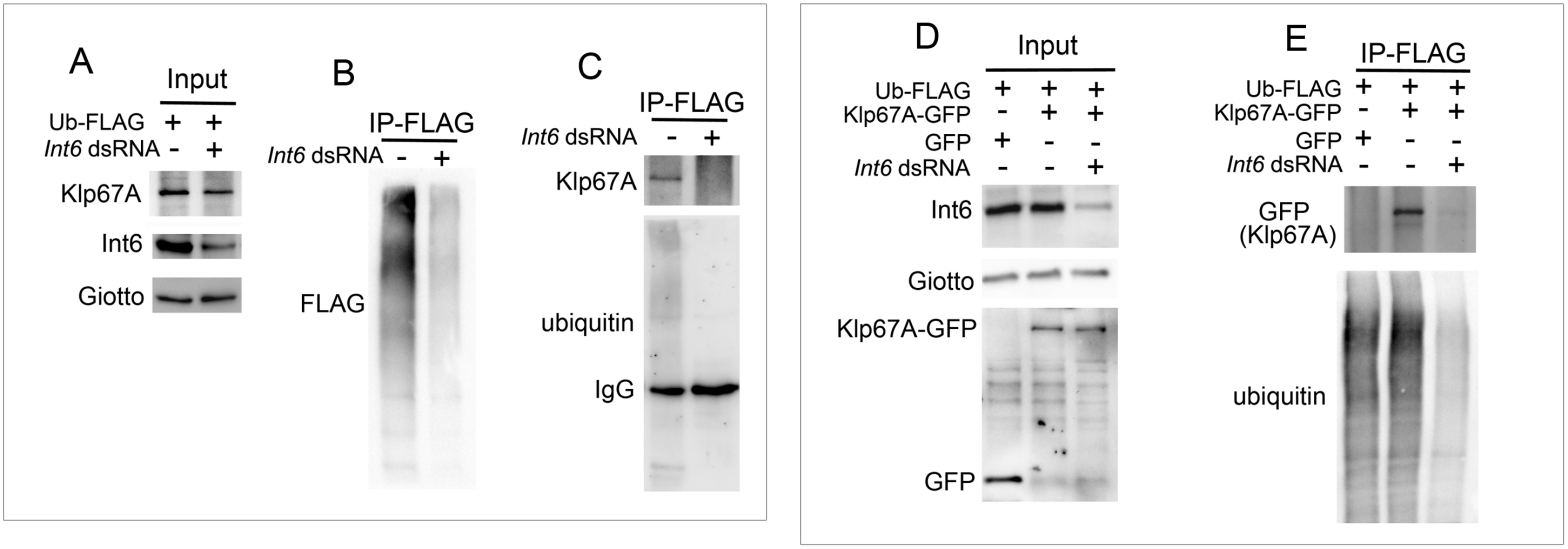
Int6 depletion reduces the level of Klp67A ubiquitination. (A-C) Int6 depletion reduces the ubiquitination level of endogenous Klp67A. Input, Giotto is a loading control (A); IP with an anti-FLAG resin showing that extracts from *Int6* RNAi cells exhibit a strong reduction in FLAG-ubiquitin-conjugated proteins (B) and reduced Klp67A ubiquitination (C) compared to controls. (D, E) Int6 depletion reduces the ubiquitination level of Klp67A-GFP. Input showing the levels of Klp67A-GFP and GFP alone in extracts from Int6-undepleted or depleted cells; Giotto is a loading control (D); IP with an anti-FLAG resin showing that in *Int6* RNAi cells there is reduction in Klp67A-GFP ubiquitination and in general protein ubiquitination compared to controls (E).

Given that Int6 interacts with both the proteasome and the signalosome, we asked whether Int6 has a role in protein ubiquitination. Because synchronization of *Drosophila* cells it is virtually impossible, we carried out our analyses using asynchronous cells populations. We performed an IP analysis using S2 cells expressing Ubiquitin-FLAG (Ub-FLAG) treated with either *Int6* dsRNA or a mock dsRNA (control). Co-IP performed using anti-FLAG agarose beads showed that precipitates from control cells exhibit a clear Klp67A band that was not detected in *Int6*-RNAi cells (Fig 10A–10C). We also performed an IP analysis using S2 cells expressing Klp67A-GFP and Ub-FLAG, and control cells expressing the GFP protein and Ub-FLAG. Western blotting analysis of precipitates obtained with anti-FLAG beads showed that *Int6* RNAi cells exhibit a substantial reduction in general protein ubiquitination compared to mock-treated cells. In addition, these precipitates displayed a very strong reduction of the ubiquitin-conjugated Klp67A-GFP band compared to precipitates from non-RNAi cells (Fig 10D and 10E). Collectively, these results indicate that Int6 mediates Klp67A ubiquitination in S2 cells.

## Discussion

### The *Int6* loss of function phenotype is largely mediated by Klp67 accumulation at kinetochores

Our results suggest that loss of Int6 leads to an accumulation of non-ubiquitinated Klp67A near the kinetochores, and that the phenotypic traits seen in Int6-deficient cells could be a consequence of this accumulation. Klp67A belongs to the kinesin 8 family and has putative orthologues in both yeasts (Kip3 in *S*. *cerevisiae* and Klp5/6 in *S*. *pombe*) and humans (Kif18A). Although these kinesins affect MT plus ends growth, they appear to act through different mechanisms. S. *cerevisiae* Kip3p acts as MT depolymerase and removes tubulin subunits from MTs in a length-dependent manner [40]. The role of mammalian Kif18A is somewhat controversial. Earlier work suggested that Kif18A, like its yeast homologue, has MT depolymerizing activity and preferentially destabilizes long microtubules [41]. However, more recent studies indicated that Kif18A suppresses addition of new tubulin subunits at MT plus ends, without directly destabilizing them [42–45].

Although the molecular mechanisms underlying Klp67A activity are currently unknown there is abundant evidence that this kinesin represses MT plus end growth. It has been reported that RNAi against *Klp67A* leads to long spindles in S2 cells [23–26], and that the kinetochore bound pool of Klp67A regulates spindle length [27]. *In vivo* analyses in S2 cells have shown that Klp67A associates with MTs and accumulates near the kinetochores; while overexpression of Klp67A leads to dose-dependent spindle shortening [26,46]. Finally, it has been reported that loss of Klp67A results in a dramatic MT elongation also in *Drosophila* embryos [47].

We have shown that Int6 depletion does not alter turnover of kinetochore MTs but leads to spindle shortening and reduction of the flux rate. Our results suggest that these phenotypes are dependent on the presence of an excess of Klp67A at the kinetochores. However, envisaging a molecular model that reconciles the three phenotypes is not straightforward. The reduction in the flux rate is consistent with a reduction in MT polymerization at plus ends, which could also lead to spindle shortening. Thus, we propose that Klp67A accumulation at the plus ends of kinetochore MTs blocks their growth without affecting their attachment and detachment rates. It is therefore possible that Klp67A caps MT plus ends and suppresses their growth just as Kif18A in human cells.

We also note that the depletion and overexpression phenotypes of Kif18A and Klp67A are rather similar. Depletion of either protein leads to long spindles, and increases anaphase chromosome velocity (in Kif18A depleted cells) or the speed MT poleward flux (in Klp67A depleted cells), which is positively correlated to the velocity of anaphase chromosome movement [23]. Overexpression of either protein leads to short spindles, reduced anaphase chromosome velocity, and increases inter-KD [42]. Thus, regardless their mechanism of action, Klp67A and Kif18A appear to have similar effects on MT plus ends during mitosis.

### Int6 depletion triggers the SAC response and causes centromere/kinetochore deformation

We have shown that in Int6-depleted cells the SAC is not satisfied even in metaphases that exhibit tightly aligned chromosomes. The precise reason for this persistent SAC activity is unclear. Studies carried out in *Drosophila* S2 cells have shown that the SAC cannot be turned off until cells achieve a sufficient intra-KD [31]. In budding yeast, SAC is active when the Mps1 Kinase phosphorylates the KNL1 orthologue Spc105, and is satisfied when Mps1 and Spc105 are separated by an internal change in kinetochore structure caused by end-on MT attachment [48]. Earlier studies in human cells suggested that SAC satisfaction requires an intra-kinetochore stretch [49]. In contrast, recent work in human cells has shown that a hyperstable kinetochore-MT attachment mediated by a non-phosphorylable form of Hec1 can silence SAC independently of the intra-KD [50,51]. In addition, it has been recently shown that taxol-treated human cells with low intra-KD can progress through mitosis if unattached kinetochores are not present [52].

Our results suggest that Int6-dependent Klp67A accumulation at kinetochores locally suppress MT growth. This would conceivably lead to a shortening of the MTs embedded into the kinetochore and low intra-KD. However, we also found that in metaphases of Int6-depleted cells the inter-KD is increased, suggesting that kinetochores are stably attached to MTs and under tension. We note that a low intra-KD accompanied by an elevated inter-KD is not an unprecedented result, as a similar effect has been observed in earlier studies. Examination of cells exposed to different treatments showed that inter-KD and intra-KD are not always correlated. For example, depletion of the condensin I subunit CAP-D2 causes a marked increase in the inter-KD and suppresses intra-KD [49]. It has been thus concluded that the intra-KD is more related to structural rearrangements within the kinetochore than to a mechanical pulling force [31,49].

We observed that Int6 depleted cells are strongly delayed in progression through metaphase by the SAC activity but eventually undergo anaphase. Thus, it appears that they can satisfy the SAC even if this takes much longer time than in control cells. In agreement with previous studies in *Drosophila* [31], it is possible that Int6-depleted cells are strongly delayed in SAC satisfaction because of their low intra-KD. However, we cannot exclude that SAC satisfaction is prevented by the centromere/kinetochore deformation, which is likely to cause internal changes in kinetochore structure that might affect SAC signaling.

The centromere/kinetochore deformation observed in Int6-depleted metaphases is both unexpected and novel. To best of our knowledge this phenotype has never been observed in any cell type. Recent work has described variations in kinetochore morphology during normal mitosis of human cells. However, these variations pertain only to proteins of the outer kinetochore domain and do not apply to metaphase kinetochores with end-on attached MTs, which are morphologically stable [53]. What is then the stimulus that induces centromere/ kinetochore deformation? Here again, we only speculate that the reduction of kinetochores MT growth caused by Klp67A accumulation could induce morphological changes in centromere/kinetochore structure.

### Int6 deficiency leads to Klp67A accumulation

Our Klp67A accumulation-based model for the Int6-dependent phenotype raises the question of the mechanism leading to reduced Klp67A ubiquitination and degradation. Int6/eIF3e interacts with both the proteasome and the COP9 signalosome (CSN), which regulates the activity of the Cullin-Ring ubiquitin Ligases (CRLs) [54]. CRLs are activated by neddylation of their cullin subunits, a process regulated by the CSN complex [54,55]. Previous studies in yeast, humans and plants have shown that depletion of Int6 homologues affects both proteasome and CSN activity [16,56]. In contrast, studies in *Drosophila* revealed that *Int6* is an essential gene required for cullin neddylation but not for proteasome function [19]. Indeed, *Int6* mutant larvae accumulate high levels of non-neddylated Cul1, while Int6 overexpression leads to accumulation of neddylated cullins [19]. Consistent with these results, we found that Int6 depletion does not result in accumulation of ubiquitinated proteins but it is instead leading to a general reduction of protein ubiquitination and to an accumulation of non-ubiquitinated Klp67A. In this respect, we would like to mention that ubiquitination is not only a way to target proteins for degradation, but it is also a widespread mechanism for conformational and functional regulation of proteins [57]. Thus, it is possible that the non-ubiquitinated Klp67A that accumulates at kinetochores of Int6-depleted cells has a slightly different activity compared to ubiquinated Klp67A or Klp67A-GFP.

As mentioned earlier, our results do not exclude the possibility that Int6-deficient cells accumulate other mitotic proteins in addition of Klp67A. However, most phenotypic traits observed in Int6-depleted cells are also seen in Klp67A overexpressing cells. Thus, if loss of Int6 results in the accumulation of another unknown protein(s) on the mitotic apparatus, this protein is unlikely to cause an appreciable mitotic defect.

### Evolutionary conservation of the Int6 function

The mitotic phenotype caused by Int6 depletion in *Drosophila* cells is quite different from the phenotype of INT6-deficient human cells, which exhibit defective spindle morphology, failure to align the chromosomes in metaphase plate and defects in chromosome segregation and cytokinesis [21]. This is not a surprise because, as mentioned previously, loss of INT6 in human cells mainly impairs proteasome activity whereas in *Drosophila* it primarily affects CSN function. However, it should be noted that Kif18A overexpression in human cells results in short spindles, compact metaphase plates and slow chromosome movement during anaphase [42,58,59], a phenotype similar to that elicited by Klp67A overexpression/Int6 downregulation. These findings suggest two hypotheses to explain why Int6-depleted *Drosophila* and human cells exhibit different mitotic phenotypes. It is possible that human INT6 does not control ubiquitin-mediated Kif18A degradation [60]. Alternatively, one might speculate that INT6-depleted human cells fail to degrade a protein(s) whose accumulation masks the phenotype caused by an excess of Kif18A.

## Materials and methods

### Cell culture and RNA interference

S2 cells were cultured at 25°C in Schneider’s medium (Sigma) supplemented with 10% fetal bovine serum (FBS, Gibco). dsRNA production and RNAi treatments were carried out according to [22]. dsRNA-treated cells were grown for 5 days at 25°C, and then processed for cytological and biochemical analyses. To depolymerize MTs, cells were treated with 25 μM colchicine (SIGMA) for 2 h. Proteasome inhibitor MG132 (10 μM; SIGMA) was added to cell cultures for 6 h.

### dsRNA production

PCR products and dsRNAs were synthesized as described in [22]. Individual *Drosophila* gene sequences were amplified by PCR from a pool of cDNAs obtained from 5 different libraries: 4 libraries from 0–4, 4–8, 8–12 and 12–24 h embryos and an imaginal disc library, all kindly provided by Nicholas H. Brown [61]. The primers used in the PCR reactions were 35 nt long and all contained a 5’ T7 RNA polymerase binding site (5’-TAATACGACTCACTATAGGGAGG-3’) joined to a gene-specific sequence. The sense and antisense gene-specific sequences were as follows: *Int6*, sense CCACCGACATTC, antisense TTGACGATCCAG; *mad2*, sense CTCTCGAAGAAC, antisense TCTATCTCGCAG; *Ndc80*, sense ATGGCAGCTTGG, antisense CGGTTAACAGGC; *Klp67A*, sense CTCATCCGGGTC, antisense ACATTCTGTTTC; *Klp10A* sense ATTGCTGTCCATC, antisense CGATCCTTGTC. The mock dsRNA used as control was obtained from an EGFP vector (Clontech) sense sequence: AGCTGTTCACCG, antisense sequence TCACGAACTCCA.

### Immunofluorescence

Preparations of S2 mitotic cells were carried out according to [22]. For Ndc80 and Klp67A immunostaining, cells were fixed for 10 min in 4% paraformaldheyde, incubated with PBS + 0.05% SDS for 30 min and then with 3% BSA in PBS for 30 min. In all the other indirect IF experiments, cells were fixed for 7 min in 3.7% formaldheyde and immunostaining was performed as described in [22] using the following antibodies, all diluted in PBS + 10% goat serum: anti-α tubulin monoclonal DM1A (1:100; Sigma); rabbit anti-Spd2 (1:3500; [62]); chicken anti-Cid (1:10000; [63]); rabbit anti-Int6 (1:100); rabbit anti-Ndc80 (1:100; a gift of M. Goldberg, Cornell University); rabbit anti-Klp67A (1:50 [27]; rabbit anti-cyclin B (1:100; [64]); rabbit anti-GFP (1:100; Torres Pines Biolabs Inc). These primary antibodies were detected by incubation for 1 h with FITC-conjugated anti-mouse (1:10, Jackson Laboratories), Cy3-conjugated anti-rabbit (1:300, Life Technologies), Cy3-conjugated anti-chicken IgGs (1:100, Jackson Laboratories), Rhodamine Red-conjugated anti-mouse (1:20, Jackson Laboratories) or FITC-conjugated anti-rabbit (1:50 Jackson Laboratories). Slides were mounted in Vectashield with DAPI (Vector) to stain DNA and reduce fluorescence fading. All images were captured using a CoolSnap HQ CCD camera (Photometrics; Tucson, AZ) connected to a Zeiss Axioplan fluorescence microscope equipped with an HBO 100 W mercury lamp. To quantify the spindle-associated Klp67-GFP, we stained preparations with both anti-GFP and anti-tubulin antibodies, which were detected by FITC-conjugated anti-rabbit and Rhodamine Red-conjugated anti-mouse, respectively. We measured the GFP and tubulin fluorescence and subtracted the background signal from each measure using the ImageJ software. We then calculated the ratio between the GFP and tubulin fluorescence.

### Measurement of inter- and intra- kinetochore distance, spindle length and centromere/kinetochore shape

The inter- and intra-kinetochore distances were measured using the calipers tool of ImageJ (NIH). For each metaphase, the inter-KD was calculated by measuring the distance between pairs of Cid signals associated with sister chromatids. The intra-KDs were calculated as [ΔNdc80-ΔCid/2], where ΔNdc80 and ΔCid are the distances between the centers of paired Ndc80 and Cid signals; for each experimental condition we analyzed at least 200 sister kinetochores/centromeres.

To assess the spindle length, metaphases were immunostained for tubulin and the centrosome marker Spd-2; we then measured the distance between the centrosomes associated with the opposite spindle poles using the calipers tools of ImageJ.

We used two different methods for measuring the extension of the Cid signals; with both methods we considered only Cid signals that were distinct from other signals. In cells where the chromosomes were tightly aligned in metaphase (mock-treated controls, MG132 treated cells, *Int6* RNAi cells, and cells overexpressing Klp67A-GFP) we directly measured the ratio between the major and minor axis of each Cid signal. We considered as major axis the one parallel or almost parallel to an ideal line orthogonal to the spindle axis (the ideal line that connects the spindle poles) bisecting the metaphase plate. Using this criterion, the axes ratio of some signal was lower than 1. To measure the extension of the signals in cells where the chromosomes were not well aligned in a metaphase plate (colchicine treated cells, *mad2* RNAi cells, *mad2 Int6* double RNAi cells, *Ndc80* RNAi cells, *Ndc80 Int6* double RNAi cells, *Klp67A* RNAi cells and *Klp67A Int6* double RNAi cells) we used the fit-ellipse function of the ImageJ software, which provided the length of the major and minor axis of each fluorescent signal.

### *In vivo* imaging

*In vivo* analysis of mitosis was performed on (i) mock-treated and *Int6* RNAi cells expressing mCherry-tubulin and histone-GFP (H2B-GFP) (a gift from Gohta Goshima), and (ii) cells expressing mCherry-tubulin and overexpressing Klp67A-GFP (see below). Cells were plated on concanavalin-A (Con-A)-treated MatTek dishes. For filming the entire mitotic process, images were taken at 1, 2 or 4 min intervals. To precisely measure the speed of chromatid movement during anaphase A, images were captured at 30–60 s intervals. 8 fluorescence optical sections were captured at 1 μm Z steps using a calibrated Prior Proscan stepping motor, with an EM-CCD camera (Cascade II, Photometrics) connected to a spinning-disk confocal head (CarvII, Beckton Dickinson) mounted on an inverted microscope (Eclipse TE2000S, Nikon). Images were acquired using Metamorph software package (Universal Imaging). Movies were made with the Metamorph software; each fluorescence image shown is a maximum-intensity projection of all sections. To calculate the velocity of chromatid-to-pole motion we divided the distance attained by the separating chromosomes sets at the end of anaphase A by the time elapsed from anaphase initiation; each measure was then divided by 2. The graph reported in Fig 1G has been obtained by averaging the velocities of 12 control and 16 *Int6* RNAi cells.

### Poleward microtubule flux and FRAP measurement

All experiments were performed using S2 cells expressing either GFP-tubulin or mCherry-tubulin. FRAP was measured in a rectangular region of interest (ROI) positioned across a half-spindle as described in [23], or within spot adjacent to a kinetochore as described in [65]. Raw intensities were background-corrected, normalized, and fitted in Matlab using the EasyFrap script [66]. Poleward flux rates in metaphase spindles were measured and analyzed according to [23].

### TEM analysis of mitosis

A suspension of cells in culture medium was centrifuged at 1000 rpm in 50 ml falcon tubes for 5 min. After removal of the supernatant, the cell pellet was immediately pre-fixed in 2.5% glutaraldehyde dissolved in the culture medium; the pellet was then gently resuspended and left in the pre-fixation solution for 15 min and gently shaken. The pre-fixed sample was next transferred to 1.5 ml Eppendorf tubes and centrifuged at 1000 rpm for 5 min. After removal of the supernatant, the pellet was fixed in a fresh 2.5% glutaraldehyde in 0.1 M sodium cacodylate buffer (pH 7.4) for 1 h at room temperature. Cells were then washed three times for 5 min each in 0.1 M sodium cacodylate buffer and post-fixed for 1 h in 1% water solution of osmium tetroxide containing few crystals of potassium ferricyanide (K_3_[Fe(CN)_6_]). After washing with three rounds of milliQ water, samples were incubated overnight at 4°C in 1% aqueous solution of uranyl acetate. On the next day, the cells were washed once with milliQ water and then dehydrated in ethanol series (30%, 50%, 70%, 96% for 10 min, and 100% for 20 min) and acetone (twice, for 20 min), and embedded in Agar 100 Resin (Agar Scientific, Essex, UK). Complete polymerization of samples was conducted by keeping them for three days in the oven at 60°C. Semi-thin sections were obtained with Reichert-Jung ultracut microtome, stained with methylene blue and analyzed with a Zeiss Axioscop 40 light microscope. Ultra-thin sections were made using Leica Ultracut ultra-microtome and stained with Reynolds lead citrate. Sections were examined with JEOL JEM-100SX transmission electron microscope at 60kV.

### Antibody generation

To obtain antibody against *Drosophila* Int6, the *Int6* sequence encoding aa 1–252 was cloned into pET200 vector (Invitrogen), and the recombinant protein was purified by electro-elution. Immunization was carried out by Agro-Bio (La Ferté St Aubin, France) according to standard protocols. The antibodies were affinity purified as described in [67].

### S2 cell transfection and overexpression

To perform co-transfections, cells were suspended in Schneider’s insect medium supplemented with 10% FBS at a concentration of 1×10^6^ cells/ml and plated, 1 ml/well, in a six-well culture dish. Each culture was inoculated with 1 μg of plasmid supplemented with Effectene transfection reagent (QIAGEN) according to the manufacturer's instructions. For selection of stably transfected cultures, cells were diluted from 1:5 to 1:10 into the appropriate selective medium 72 h after transfection. For generating stable cell lines we used the following plasmids: H2B-GFP and mCherry- tubulin (both gifts from Gohta Goshima, Nagoya University, Japan), pMT-Klp67A-GFP (a gift from Ronald D. Vale, UCSF, CA), pCoHygro and pCoBlast (both from Invitrogen). pMt-Klp67A-GFP expression was induced with 10 μM CuSO_4_ for 12 h (for biochemical experiments) or 100 μM CuSO_4_ for 48 h (for IF experiments). For transient expression we used p-AWG (from DGRC, Indiana University, Bloomington) and a FLAG-tagged ubiquitin expressing plasmid generated in our laboratory (the structure of this plasmid can be schematized as follows: pJZ4-Kpn1-6His-Xpress-FLAG-ubiquitin-Xba-pJZ4).

### Western blotting and ubiquitination assays

For immunoblotting of *Drosophila* proteins, S2 cells were washed in cold PBS and homogenized in lysis buffer (50 mM Hepes KOH pH 7.6, 1 mM MgCl2, 1mM EGTA, 1% Triton X-100, 45 mM NaF, 45 mM β-glycerophosphate, 0.2 mM Na3VO4) in the presence of a cocktail of protease inhibitors (Roche). Cell extracts were pelleted at 15,000 g in an Eppendorf centrifuge for 15 min at 4°C and the supernatants were analyzed by Western blotting according to [68], using the following antibodies, all diluted in TBS-T (TBS with 0.1% Tween 20): rabbit anti-Klp67A (1:500; [27]); rabbit anti-Int6 (1:1000); rabbit anti-Giotto (1:5000; [69]); anti-α tubulin monoclonal DM1A (1:1000, Sigma); rabbit anti-GFP (1:2500; Torrey Pines Biolabs Inc); rabbit anti-cyclin B (1:1000; [64]); mouse anti-ubiquitin (1:1000; Covance); anti-Flag-HRP-conjugated (1:3000, Invitrogen). These primary antibodies (except the anti-Flag-HRP-conjugated) were detected using HRP conjugated anti-mouse and anti-rabbit IgGs and the ECL detection kit (all from GE Healthcare). Band intensities were quantified by densitometric analysis with Image Lab software (Bio-Rad).

For the *in vivo* ubiquitination assay, S2 cell were transfected with p6His-Xpress-FLAG-ub, pAWG and pMt-Klp67A-GFP and after 72 h treated with *int6* dsRNA as described above. Expression of pMt-Klp67A-GFP was induced by an overnight treatment with 10 μM CuSO_4_. Cells were harvested and lysed with lysis buffer (50 mM Tris [pH 7.5], 120 mM NaCl, and 0.5% NP40) containing 1% (w/v) sodium dodecyl sulfate (SDS) that was preheated to 100°C [70]. Before binding to the anti-Flag beads, NaCl and SDS concentration in the binding buffer were adjusted to 500 mM and 0.1%, respectively. After pull-down, the beads were washed with lysis buffer containing 0.1% SDS and were used for immunoblot analysis. Blots were imaged with the ChemiDoc MP imager (Bio-Rad); band intensities were quantified using Image Lab software (Bio-Rad).

## Supporting information

S1 Fig Anaphase and telophase spindles of Int6-depleted cells are shorter than those of control cells. (A, B) Examples of control (A), and Int6-depleted S2 cells stained for DNA (DAPI) and tubulin. (C) Spindle length of metaphase, anaphase and telophase figures from control and *Int6* RNAi cells. Differences in length are all significant with p < 0.001 (Student's t test).
(TIF)

S2 Fig Int6 depletion does not affect microtubule turnover near the spindle poles. (A) Fluorescence recovery of α tubulin-GFP near the spindle poles in mock-treated cells and *Int6* RNAi cells. Squares denote the bleached region; numbers refer to seconds after photobleaching. (B) Averaged curves and recovery parameters.
(TIF)

S3 Fig Spindle shortening during prometaphase/metaphase of Klp67A-GFP overexpressing cells. The spindle length variation with time in Klp67A-GFP overexpressing cells is compared with those of Int6-depleted and control cells; error bars indicate SEM.
(TIF)

S4 Fig Klp67A-GFP overexpression does not affect the fluorescence recovery time of kinetochore-associated MTs. (A-B) Averaged curves and recovery parameters near chromosomes-associated mCherry-marked MTs in cells showing low or no expression of Klp67A-GFP (A) or overexpressing Klp67A-GFP (B).
(TIF)

S5 Fig Int6 and Klp10 have antagonistic roles in spindle length regulation. (A) Examples of metaphase and anaphase spindles in *Klp10A* RNAi cells (top panels) and *Klp10A Int6* double RNAi cells (bottom panels). (B, C) Mitotic parameters (B) and average spindle length (C) in mock-treated, *Int6* RNAi, *Klp10A* RNAi, and *Klp10A Int6* double RNAi cells. ***, significant with p < 0.0001 in the Student’s t rest.
(TIF)

S6 Fig Localization of Int6 in S2 dividing cells. (A) Cells stained for DNA (blue), tubulin (green) and Int6 (red). Note the modest Int6 enrichment around the chromosomes of the metaphase figure of mock-treated cells and lack of immunostaining of the metaphase from *Int6* RNAi cells. (B) Entire Western blotting showing the specificity of the anti-Int6 antibody.
(TIF)

S1 Movie Mitosis in control S2 cells expressing histone-GFP and mCherry tubulin. (MOV)

S2 Movie Prolonged metaphase arrest in Int6-depleted S2 cells expressing histone-GFP and mCherry tubulin. (MOV)
