# Supplementary figures and images for: The Drosophila orthologue of the INT6 onco-protein regulates mitotic microtubule growth and kinetochore structure
Source: PLoS Genet. 2017 May 15;13(5):e1006784. doi: 10.1371/journal.pgen.1006784 (PMC5448806; doi:10.1371/journal.pgen.1006784)

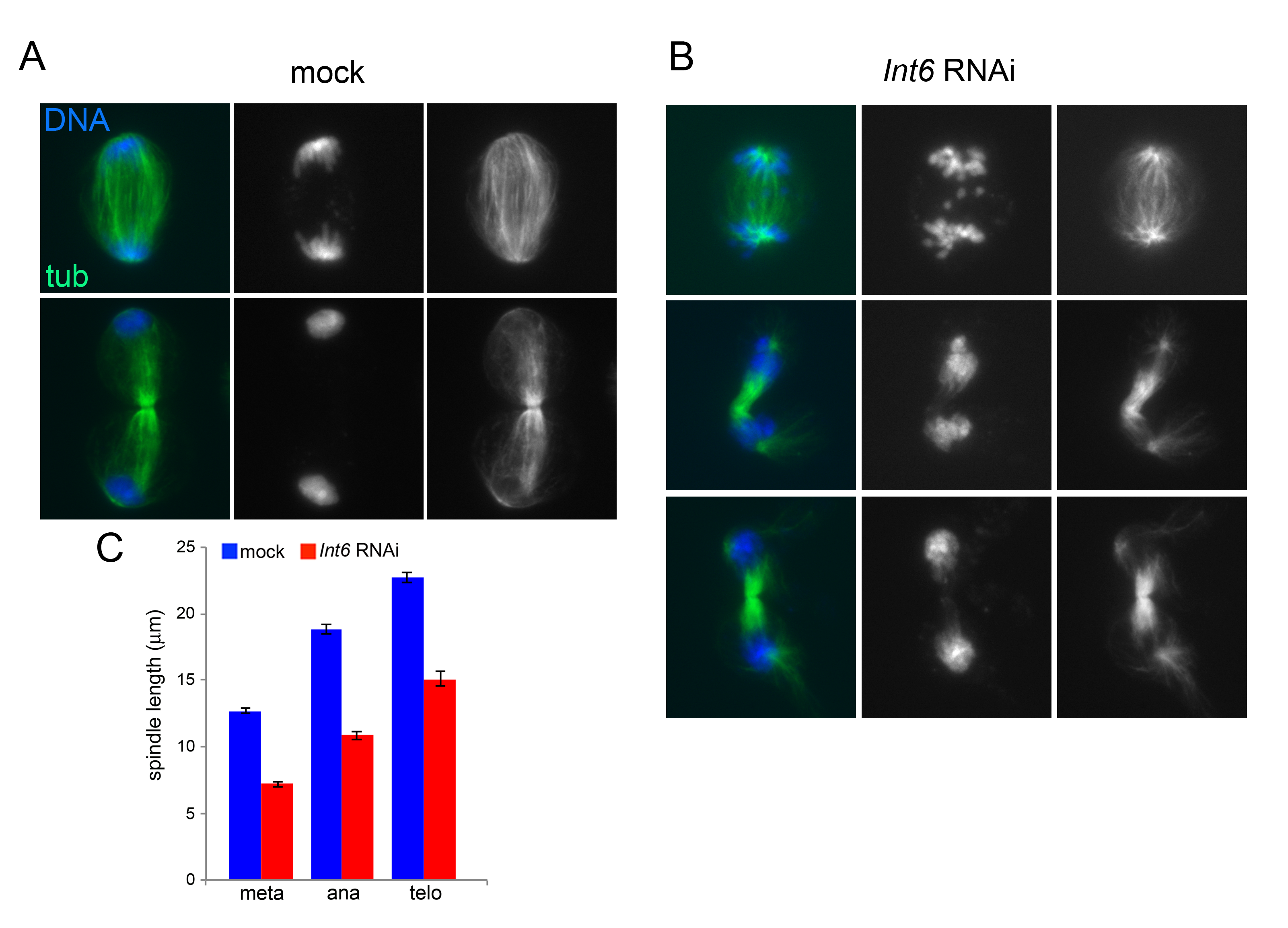

Supplement: S1 Fig — (A, B) Examples of control (A), and Int6-depleted S2 cells stained for DNA (DAPI) and tubulin. (C) Spindle length of metaphase, anaphase and telophase figures from control and Int6 RNAi cells. Differences in length are all significant with p < 0.001 (Student's t test). (TIF) [file pgen.1006784.s001.tif]

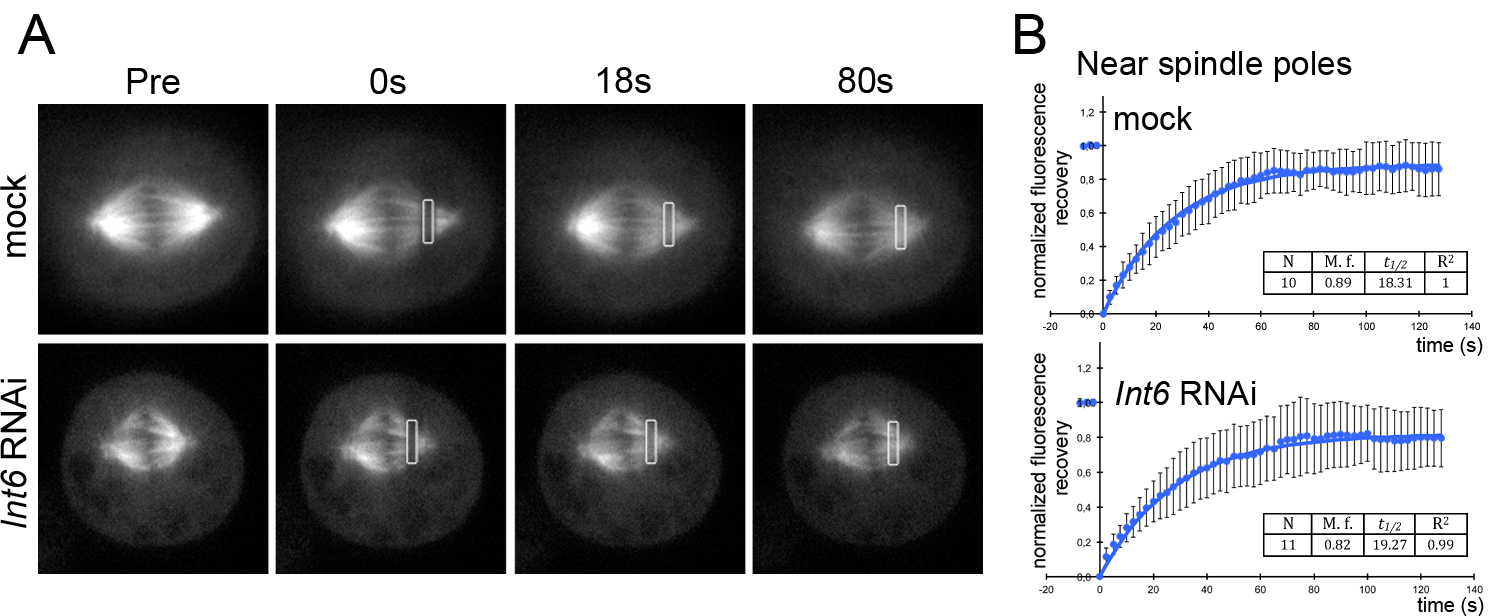

Supplement: S2 Fig — (A) Fluorescence recovery of α tubulin-GFP near the spindle poles in mock-treated cells and Int6 RNAi cells. Squares denote the bleached region; numbers refer to seconds after photobleaching. (B) Averaged curves and recovery parameters. (TIF) [file pgen.1006784.s002.tif]

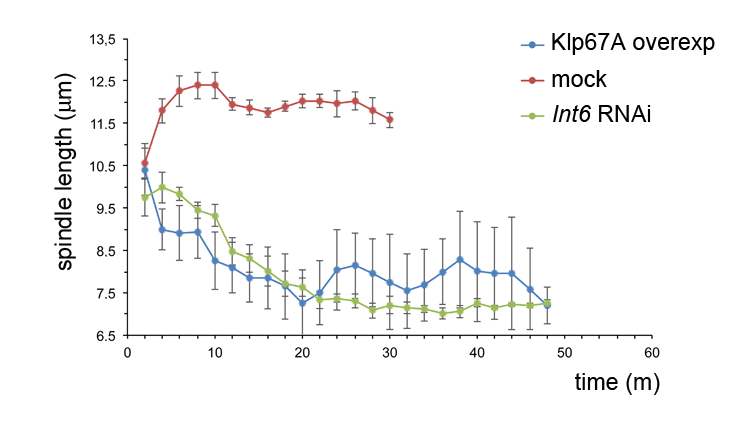

Supplement: S3 Fig — The spindle length variation with time in Klp67A-GFP overexpressing cells is compared with those of Int6-depleted and control cells; error bars indicate SEM. (TIF) [file pgen.1006784.s003.tif]

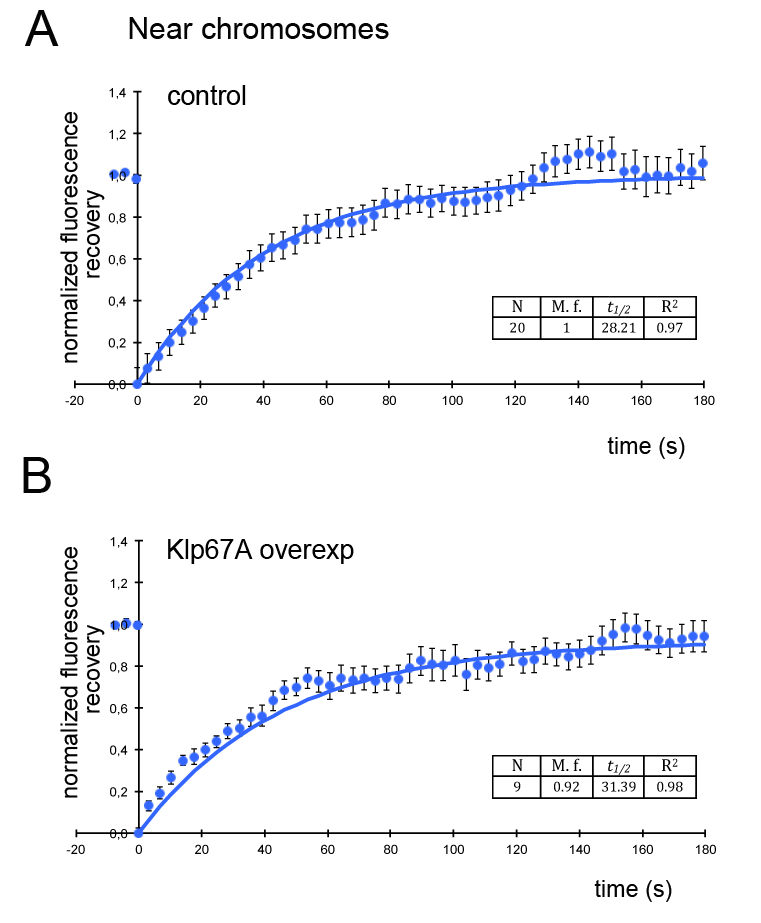

Supplement: S4 Fig — (A-B) Averaged curves and recovery parameters near chromosomes-associated mCherry-marked MTs in cells showing low or no expression of Klp67A-GFP (A) or overexpressing Klp67A-GFP (B). (TIF) [file pgen.1006784.s004.tif]

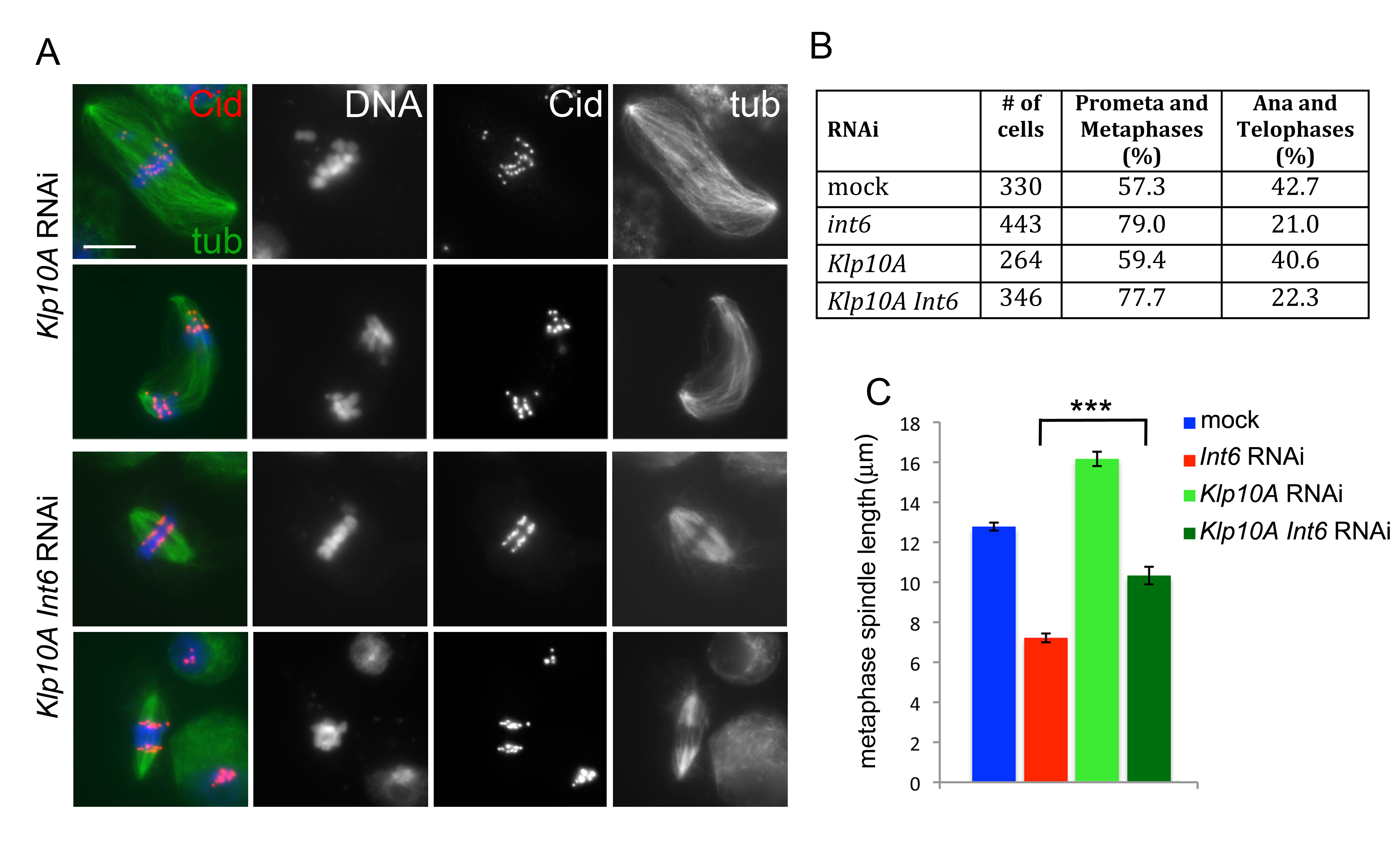

Supplement: S5 Fig — (A) Examples of metaphase and anaphase spindles in Klp10A RNAi cells (top panels) and Klp10A Int6 double RNAi cells (bottom panels). (B, C) Mitotic parameters (B) and average spindle length (C) in mock-treated, Int6 RNAi, Klp10A RNAi, and Klp10A Int6 double RNAi cells. ***, significant with p < 0.0001 in the Student’s t rest. (TIF) [file pgen.1006784.s005.tif]

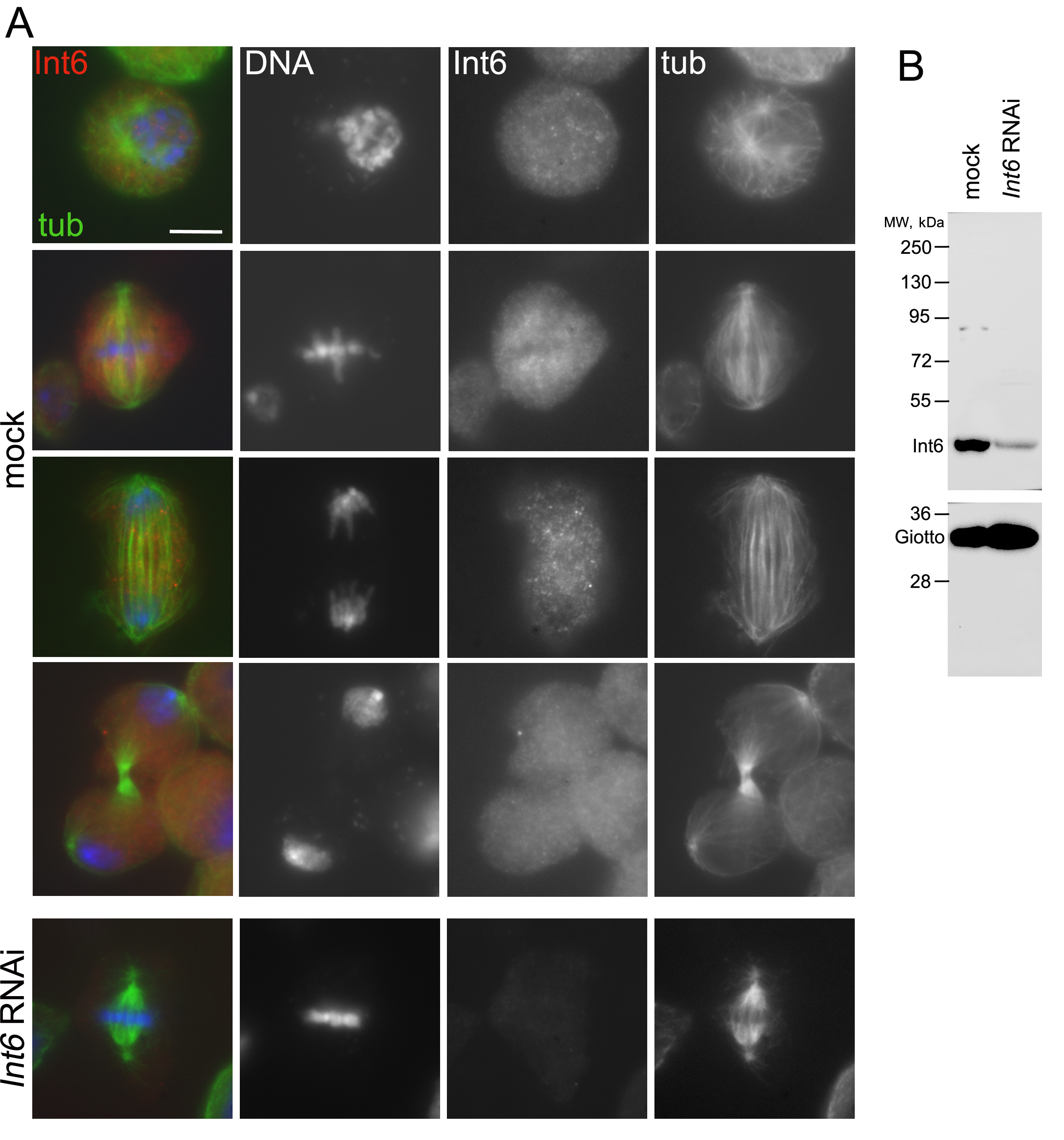

Supplement: S6 Fig — (A) Cells stained for DNA (blue), tubulin (green) and Int6 (red). Note the modest Int6 enrichment around the chromosomes of the metaphase figure of mock-treated cells and lack of immunostaining of the metaphase from Int6 RNAi cells. (B) Entire Western blotting showing the specificity of the anti-Int6 antibody. (TIF) [file pgen.1006784.s006.tif]
